# The Sisal Virome: Uncovering the Viral Diversity of *Agave* Varieties Reveals New and Organ-Specific Viruses

**DOI:** 10.3390/microorganisms9081704

**Published:** 2021-08-10

**Authors:** Gabriel Quintanilha-Peixoto, Paula Luize Camargos Fonseca, Fábio Trigo Raya, Marina Pupke Marone, Dener Eduardo Bortolini, Piotr Mieczkowski, Roenick Proveti Olmo, Marcelo Falsarella Carazzolle, Christian A. Voigt, Ana Cristina Fermino Soares, Gonçalo Amarante Guimarães Pereira, Aristóteles Góes-Neto, Eric Roberto Guimarães Rocha Aguiar

**Affiliations:** 1 Institute of Biological Sciences, Universidade Federal de Minas Gerais, Belo Horizonte 31270-901, Brazil; gabrielqpx@ufmg.br (G.Q.-P.); camargos.paulaluize@gmail.com (P.L.C.F.); gigatonn@gmail.com (D.E.B.); Roenick@gmail.com (R.P.O.); 2 Department of Genetics and Evolution, Institute of Biology, Universidade Estadual de Campinas, Campinas 13083-872, Brazil; fabioraya@gmail.com (F.T.R.); marina.marone@gmail.com (M.P.M.); mcarazzo@unicamp.br (M.F.C.); goncalo@unicamp.br (G.A.G.P.); 3 High-Throughput Sequencing Facility, School of Medicine, University of North Carolina at Chapel Hill, Chapel Hill, NC 27516, USA; Piotr_Mieczkowski@med.unc.edu; 4 CNRS UPR9022, INSERM U1257, Université de Strasbourg, 67084 Strasbourg, France; 5 BASF Innovation Center Gent, 9052 Gent, Belgium; christian.voigt@basf.com; 6 Center of Agricultural, Environmental and Biological Sciences, Universidade Federal do Recôncavo da Bahia, Cruz das Almas 44380-000, Brazil; acsoares@ufrb.edu.br; 7 Center of Biotechnology and Genetics, Department of Biological Science, Universidade Estadual de Santa Cruz, Ilhéus 45662-900, Brazil

**Keywords:** virome, metatranscriptomics, *Agave*

## Abstract

Sisal is a common name for different plant varieties in the genus *Agave* (especially *Agave sisalana*) used for high-quality natural leaf fiber extraction. Despite the economic value of these plants, we still lack information about the diversity of viruses (virome) in non-*tequilana* species from the genus *Agave*. In this work, by associating RNA and DNA deep sequencing we were able to identify 25 putative viral species infecting *A. sisalana, A. fourcroydes,* and *Agave* hybrid 11648, including one strain of *Cowpea Mild Mottle Virus* (CPMMV) and 24 elements likely representing new viruses. Phylogenetic analysis indicated they belong to at least six viral families: Alphaflexiviridae, Betaflexiviridae, Botourmiaviridae, Closteroviridae, Partitiviridae, Virgaviridae, and three distinct unclassified groups. We observed higher viral taxa richness in roots when compared to leaves and stems. Furthermore, leaves and stems are very similar diversity-wise, with a lower number of taxa and dominance of a single viral species. Finally, approximately 50% of the identified viruses were found in all Agave organs investigated, which suggests that they likely produce a systemic infection. This is the first metatranscriptomics study focused on viral identification in species from the genus *Agave*. Despite having analyzed symptomless individuals, we identified several viruses supposedly infecting *Agave* species, including organ-specific and systemic species. Surprisingly, some of these putative viruses are probably infecting microorganisms composing the plant microbiota. Altogether, our results reinforce the importance of unbiased strategies for the identification and monitoring of viruses in plant species, including those with asymptomatic phenotypes.

## 1. Introduction

Sisal is a common name for different species and hybrid varieties in the genus *Agave* (especially *Agave sisalana*) cultivated worldwide for the production of hard natural fibers [1,2]. In Brazil, the greatest producer of sisal fibers, sisal-producing areas are concentrated in the Northeastern region of the country, especially in the state of Bahia [3], followed by the states of Paraíba and Pernambuco [4]. The semi-arid portion of this region, known as the *Caatinga* biome, bears a high richness of highly adapted species, in all the domains of life [5,6]. Although the cultivated *Agave* species are not native to that region, they are adapted to the specific environmental conditions of this area, including low rainfall, high temperatures, and low aboveground biomass coverage [7]. These traits of resistance to abiotic stresses make sisal one of the few cultivation options available in the *Caatinga* biome, historically neglected in infrastructure projects.

Viral infections in plants may cause damage to specific structures, such as the photosynthetic apparatus in the leaves [8], the roots system [9], and also in growth and development, as in early flowering, often used to accelerate yielding [10]. In sisal, these forms of damage could harm fiber quality, which is the main commercial and valuable trait in these *Agave* species. Studies in other economically important plants, such as grapevine, revealed that the viral diversity goes beyond pathogenic species, which broadens our knowledge of plant viruses [11,12]. Therefore, the study of neglected cultures could provide not only a better perspective of viral diversity in economically relevant individuals and related species but also a glimpse of the real circulation of viruses in the region where it is grown.

Sisal and other plants, as all known domains of life, are susceptible to viral infection. Nonetheless, the knowledge on viruses infecting non-*tequilana* species in the genus *Agave* is very scarce and restricted to low-throughput strategies, with only a few published articles describing infections on sisal until this date. Pinkerton and Bock (1969) [13] indicated (but did not confirm) viruses as the causative agent of the parallel streak of sisal in Kenya; Galvez et al. (1977) [14] described the *Necrotic streak virus* in the genus *Furcraea* (not to be confused with *Agave fourcroydes*, one of the plant taxa in the present study), later described in-depth by Morales et al. (1992) [15]; and Izaguirre-mayoral et al. (1995) [16], which described the infection of *Cactus X virus* on *A. sisalana*. In a more recent record, Chabi-Jesus et al. (2019) [17] described the presence of the *Citrus Chlorotic Spot virus* in *Agave desmettiana* individuals. Hence, the real diversity of viruses associated with sisal and its respective associated In these cases, different metagenomics strategies have been applied, including small RNA (sRNA) sequencing [18,19,20,21], DNA sequencing [22], simultaneous extraction of microorganisms and surroundings is still unclear. As mentioned before, different works in other plant species show an abundant viral diversity, such as those observed in lilies [20], grapevines [23], and peaches [24], including those of viruses infecting plant-associated microorganisms [25,26]. DNA and RNA followed by digestion with RNases/DNases [27], and even amplification-based methods [28]. Among them, the next-generation sequencing of RNA (metatranscriptomics) has been consolidated as an important unbiased strategy for virome studies [23,29,30]. Indeed, this strategy allows the detection of almost all viral species, since most of the viruses are made of or produce RNA molecules during replication, which would not be detected using DNA deep sequencing.

Therefore, our study focused on uncovering the viral diversity of *Agave* species using metatranscriptomics. We investigate three different *Agave* cultivars used for fiber extraction (*A. fourcroydes*, *A. sisalana* and *Agave* Hybrid 11648) collecting samples from three different organs (leaves, stems, and roots) in biological triplicates. Using this approach in association with DNA deep sequencing to filter out endogenous elements, we identified 25 putative viral species in asymptomatic *Agave* individuals, including the known species *Cowpea Mild Mottle Virus* (CPMMV) and 24 previously unknown viral species, belonging to at least six viral families: *Alphaflexiviridae*, *Betaflexiviridae*, *Botourmiaviridae*, *Closteroviridae*, *Partitiviridae*, *Virgaviridae* and three distinct unclassified viruses. *A. fourcroydes* displayed the highest diversity among the three *Agave* taxa while the roots were the plant organ with the highest viral diversity. We also observed discrepant abundance for the same virus among different organs, highlighting replication strategy preferences, such as observed for *Sisal-associated Closterovirus A* (higher in leaves and stems) and *Sisal-associated Virgavirus C* (higher in roots). Altogether, our results highlight both the importance of unbiased high-throughput strategies for the discovery of new viral species and also the relevance of screening asymptomatic plants for obtaining a more realistic viral diversity scenario.

## 2. Materials and Methods

### 2.1. Sample Origin, Transcriptome Sequencing, Assembly, and Quantification

**Figure 1:**
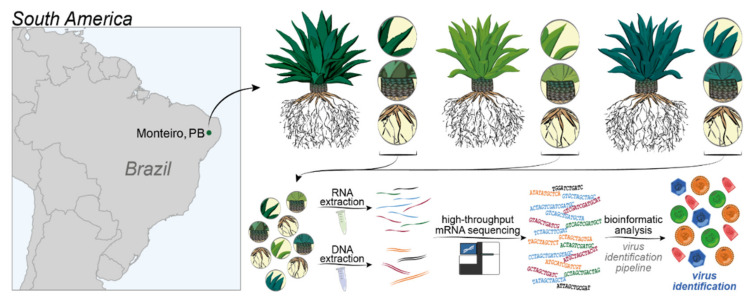
Strategy applied to uncover the virome of *Agave* species. The dot in the map indicates the localization of Monteiro, in the state of Paraíba. *Agave fourcroydes*, *Agave sisalana* and Agave Hybrid 11648 (and their respective organ samples) are indicated in different tones of green, for differentiation purposes. The following methods are indicated in the lower portion of the figure; RNA extraction, DNA extraction, high-throughput sequencing in the Illumina platform, and bioinformatics analysis (see further details in the Material and Methods section).

In this study, we investigated the sisal virome using transcriptomic datasets previously generated by Raya et al. (2020) [29]. Our samples were extracted from sections of the leaves, stems, and roots from three different seven-year-old adult plants of *A. sisalana, A. fourcroydes*, and the *Agave* hybrid 11648 ((*A. amaniensis x A. angustifolia*) *x A. amaniensis*) collected at the EMBRAPA collection in Monteiro, in the state of Paraíba, Brazil (07°53′ S; 37°07′ W, elevation: 619 m). For all cultivars, leaves (central fraction of the proximal-distal axis), roots, and stems were sampled from seven-year-old healthy adult plants. For each cultivar, three biological replicates growing side-by-side were sampled. To maintain leaf maturity equivalency within the cultivars, we sampled the fifth leaf counted from the central spike of each plant. Although the plants were maintained in the field and exposed to long drought periods typical of the Caatinga biome, all the collected individuals were healthy, with the leaves showing homogenous green coloration, with no visible symptoms of diseases (necrosis, chlorosis, spots, etc.). Total RNA was extracted according to the protocol described by Zeng and Yang, (2002), with the modifications proposed by Le Provost et al. (2003) [30]. mRNA library preparation and sequencing were done at the High-Throughput Sequencing Facility of the Carolina Center for Genome Sciences (University of North Carolina at Chapel Hill, NC, USA). The libraries were prepared using the KAPA Stranded mRNA-Seq kit (07962193001) for Illumina platforms following the manufacturer’s protocol, using 1 µg total RNA. The sequencing was done on the Illumina HiSeq 4000 system, generating 50 bp paired-end reads. More details can be found in Raya et al. (2020) [29]. The DNA Libraries, one for each *Agave* taxon, were prepared using the KAPA Hyper Prep Kit (07962312001) following the manufacturer’s instructions. For the library preparation, 1000 ng of fragmented DNA (average size ~280 bp) was used. Subsequently, the three DNA libraries were pooled together and sequenced with the Illumina HiSeq 4000 system. The transcriptome was de novo assembled using Trinity v. 2.5.1 for each species separately [31]. Transcript quantification was performed using kallisto v 0.44.0 [32], and ORF prediction was carried out with TransDecoder v. 5.0.2 [33]. An overview of the methods can be found in Figure 1.

### 2.2. Identification of Virus-Derived Sequences

Sequence similarity searches were performed similarly for all the *Agave* transcriptomes. Assembled transcripts were aligned to the nucleotide (NT) database of GenBank (available online: ftp://ftp.ncbi.nlm.nih.gov/blast/db/, accessed on 14 April 2020) using BLASTn [34], keeping only the best hit (with the -max_target_seqs flag set to 1) for each sequence. Viral hits were used to identify transcripts possibly derived from viruses present in the samples. The closest reference viral genomes whose transcripts displayed similarity at nucleotide level were obtained from GenBank and used to align the transcripts back using BLASTn to assess genome coverage. To create coverage plots, those transcripts were sampled with *sample.py* (available on Supplementary Materials) and aligned to a full viral genome with Bowtie2 [35], and, subsequently, formatted with Samtools [36] and visualized with IGV [37] and Mauve [38]. The plant variety containing the largest number of transcripts aligned to the reference viral genome was chosen to obtain a full consensus-based genome. Pre-processed reads were aligned to the reference viral genome with Bowtie2 as previously described, and the output SAM file was analyzed with both Samtools and BCFtools to generate a consensus sequence in FASTA format file representing the reconstituted viral genome.

Contigs that did not exhibit significant similarity with reference genomes in public databases, at the nucleotide level (BLASTn requiring *e*-value <1×10^−5^ against NCBI NT database), were compared against the non-redundant (NR) database of GenBank (available online: ftp://ftp.ncbi.nlm.nih.gov/blast/db/, accessed on 14 April 2020) with the BLASTx module of Diamond [39], and viral hits were selected considering E-value < 1×10^−3^. Contigs from different libraries that displayed matching regions with the same viral sequences were submitted to redundancy removal and contig extension using CAP3 [40]. Non-redundant contigs were further analyzed to investigate possible conserved domains using HMMER [41].

### 2.3. Manual Curation and Detection of Endogenous Viral Elements

In order to distinguish whether viral sequences were derived from an endogenous or exogenous origin, we sequenced three (one for each *Agave* taxa) DNA libraries from the leaves of the same individuals from which we produced the transcriptomes. Genomic DNA was prepared using the KAPA Hyper Prep Kit (07962312001) following the manufacturer’s instructions. For the library preparation, 1 ug of fragmented DNA with an average length of 280 bp was used as input material, and then four PCR cycles were used for amplification. Libraries were sequenced with Illumina HiSeq 4000 platform Finally, to investigate the origin of possible viral sequences, raw genomic reads were aligned to the candidate viral genomes using Bowtie2 allowing zero mismatches. Removal of false-positive results was adapted from Aguiar et al. (2015) [42]. Briefly, we removed sequences presenting similarity with retroviral elements, containing truncated ORFs and viral transcripts assigned to non-retroviral families that presented alignment from DNA sequencing libraries (at least 10 reads covering 70% sequence) were considered derived from endogenous viral elements (EVEs).

### 2.4. Phylogenetic Analysis

A classification-based method was developed to stipulate the confidence levels of the detected species, and those sequences suitable for phylogenetic analysis. From lower confidence to higher confidence: Class 0 would comprise viral species with no RNA-dependent RNA polymerase (RdRp) sequence detected, Class 1 would comprise elements in which a partial RdRp sequence (<500 bp) was found accompanied or not by other viral proteins, Class 2 would encompass viruses with large fragments (>500 bp) of RdRp, while Class 3 would consist of viral species containing a large fragment (>500 bp) of RdRp and some other viral protein (except for the coat protein). Finally, Class 4 consists of viral species with a large fragment (>500 bp) of RdRp and the coat protein detected, with the facultative presence of other viral proteins. The species classified within Classes 2–4, were then selected for phylogenetic analysis. Then, assembled contigs that showed similarity with RdRp were translated into amino acid sequences and aligned with related viral sequences available in NCBI public protein database [43] using MAFFT [44]. The accession numbers of the viral sequences used in this study are listed in Table S1. The protein best-fit model was selected for each alignment file using ProtTest 3.2, considering the Akaike information criterion (AIC) [45]. Maximum likelihood (ML) trees were constructed in MEGA X [46] with 1000 bootstrap replicates for evaluating branch support. The trees were mid-point rooted and edited in FigTree (available online: http://tree.bio.ed.ac.uk/software/figtree/, accessed on 14 April 2020) and Geneious Prime 2020 1.2. (available online: https://www.geneious.com, accessed on 14 April 2020).

### 2.5. Diversity Analysis

The quantification metrics for selected viral contigs (in tpm—transcripts per million) were obtained from kallitsto [32]. The resulting expression matrix was analyzed with the packages *vegan* and *vegetarian* from R [47], while PCA analysis and diversity indices were produced with PAST [48] using as variables the viral expression of our 25 species on the three analyzed organs (leaves, stems, and roots) for each of the three *Agave* taxa. For the PCA analysis, quantitative data from kallisto were used, while for the diversity indices, a presence-absence matrix was generated.

## 3. Results

### 3.1. Identification of Virus-Derived Sequences

**Figure 2:**
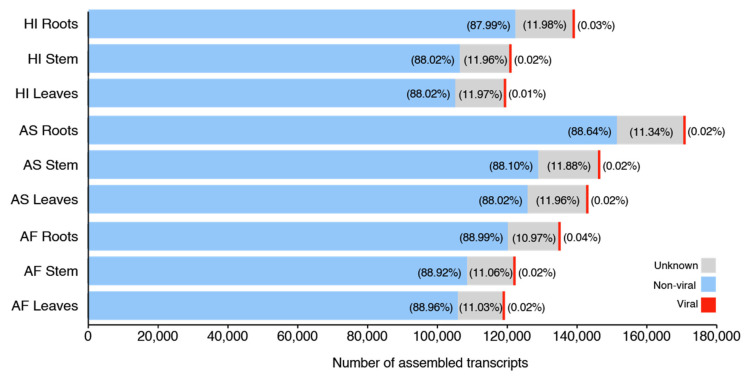
Overview of sequence similarity searches. The bar plots indicate the non-normalized values of viral, unknown, and other (non-viral) contigs in the nine analyzed samples. Color keys are indicated in the figure. Percentages are indicated inside color bars or on the right side, for viral contigs values. “Unknown” represents sequences with no significant hit at NCBI nucleotide and protein databases, while “non-viral” indicates sequences with hits with any other organism but viral.

In this study, we took advantage of RNA deep sequencing to identify and characterize the virome of *Agave* taxa. We deep sequenced 27 RNA libraries from two plant species, and a hybrid variety (three plant taxa × three organs × three replicates for leaf, stem, and root tissues), totalizing 559,267,611 raw reads (Table S2). Transcriptome assemblies produced 251,953 contigs, considering all libraries. Sequence similarity searches revealed that the percentage of viral sequences (not considering contig extension with CAP3) represented ~0.02% of all transcripts assembled for almost all the organs and varieties, with exception of the roots of *Agave fourcroydes* (0.04%) and *Agave* Hybrid 11648 (0.03%) and the leaves of *Agave* Hybrid 11648 (0.01%) (Figure 2). From the total, 10 contigs showed similarity with viruses at the nucleotide level (Tables S3 and S4), and 80 contigs (extended with CAP3) showed similarity with viral sequences at amino acid level, compared against NCBI sequence databases NT and NR, respectively (Tables S3 and S5). These initial 90 viral contigs identified by sequence similarity suggested the presence of 28 viral species associated with the sisal samples, including one known species (CPMMV) and 27 new viral species, which were assigned to at least seven viral families; Alphaflexiviridae (three species, four contigs), Betaflexiviridae (seven species, 22 contigs), Botourmiaviridae (two species, two contigs), Closteroviridae (one species, eight contigs), Mitoviridae (one species, five contigs), Partitiviridae (one species, one contig), Virgaviridae (three species, 19 contigs), and Unclassified species including Unclassified dsRNA (four species, 12 contigs), Unclassified Riboviria (one species, two contigs), or simply Unclassified (four species, 13 contigs). Details about sequence similarity searches can be visualized in Table S5.

### 3.2. Manual Curation and Detection of Endogenous Viral Elements

In our analysis, we took different precautions to avoid the occurrence of false-positive sequences, a major problem in virome studies, which usually corresponds to either poorly assembled/annotated sequences and/or endogenous viral elements (EVEs) (detailed in methods). Firstly, we sequenced genomic DNA from all three plant taxa to verify if any of our contigs assigned to viruses would be derived from EVEs. We also analyzed top similarity hits considering the online version of NCBI Blast (which uses the most updated versions of amino acid databases), the ORF profile, the existence of conserved domains, the contig length, and the presence of proteins encoding to polymerase and coat proteins.

Our manual curation discarded 13 contigs initially detected as viral, which were named with the prefix *disc* plus a number for identification (Table S6), three of which (*disc1, disc2, and disc3,* showing similarity with the RdRp of *Macrophomina phaseolina* *tobamo-like virus* (*disc1*) and the putative proteins P31 and P33 of *Pistachio ampelovirus A* (*disc2* and *disc3*), respectively). These transcripts presented counts in the genomic DNA sequencing and/or the presence of non-viral domains. One contig was discarded based solely on the detection of non-viral protein domains (originally showing similarity with a movement protein of *Podosphaera prunicola tobamo-like virus*), and the three remaining contigs with neither protein domains nor hits with viral sequences using the web version of NCBI BLASTx (originally showing similarity with a hypothetical protein of *Aspergillus foetidus* *dsRNA mycovirus*, and the RdRp of *Aspergillus* *mycovirus 341* and *Helicobasidium mompa* partitivirus V1-2). Five other contigs putatively derived from mitoviruses displayed counts on DNA sequencing and were also discarded. Twenty-four possible new viral species were left after false-positive results were removed. To stipulate the confidence levels of the species detected in our analyses, we developed a classification-based system consisting of five classes and used this method to select species for further phylogenetic characterization (Table S7). From lower confidence to higher confidence: Class 0 comprises viral species with no RdRp sequence detected (two viral species), Class 1 comprises elements in which a partial RdRp sequence (<500 bp), found accompanied or not by other viral proteins (10 viral species), Class 2 encompasses viruses with large fragments (>500 bp) of RdRp (eight viral species), while Class 3 consists of viral species containing a large fragment (>500 bp) of RdRp and some other viral protein (except for the coat protein) (one viral species), and finally, Class 4 is made up of viral species with a large fragment (>500 bp) of RdRp and the coat protein detected, with other viral proteins being facultative (four viral species) (Table S7). The species classified within Classes 2–4 (13 viral species) were selected for phylogenetic analysis.

### 3.3. Reconstitution of Cowpea Mild Mottle Virus (CPMMV) Strain Associated with Agave Species

Sequence similarity searches revealed 10 contigs showing high similarity and identity with CPMMV at the nucleotide level, of which one of them (assembled from *Agave fourcroydes*) was 3600 nt-long. Thus, we decided to apply a reference-based strategy to reconstruct the whole genome of this *Agave*-derived lineage (Figure S1, Tables S3 and S4) taking advantage of the libraries derived from *Agave fourcroydes* to obtain the full viral genome of the sisal isolate of CPMMV. As seen in Figure S2, raw RNA reads covered 100% of the closest reference genome, with some genetic variability between the reference and our isolate, which probably reflects the real variation between strains but did not impact the structural annotation of our CPMMV strain (Table S4, Figure S3). The full genome sequence (8193 nt) of the sisal *Cowpea mild mottle virus* isolate PB:AF (*Carlavirus*, Betablexiviridae) is available on GenBank under the accession code *MZ329767*.

### 3.4. Phylogenetic Characterization of New Viral Sequences

**Figure 3:**
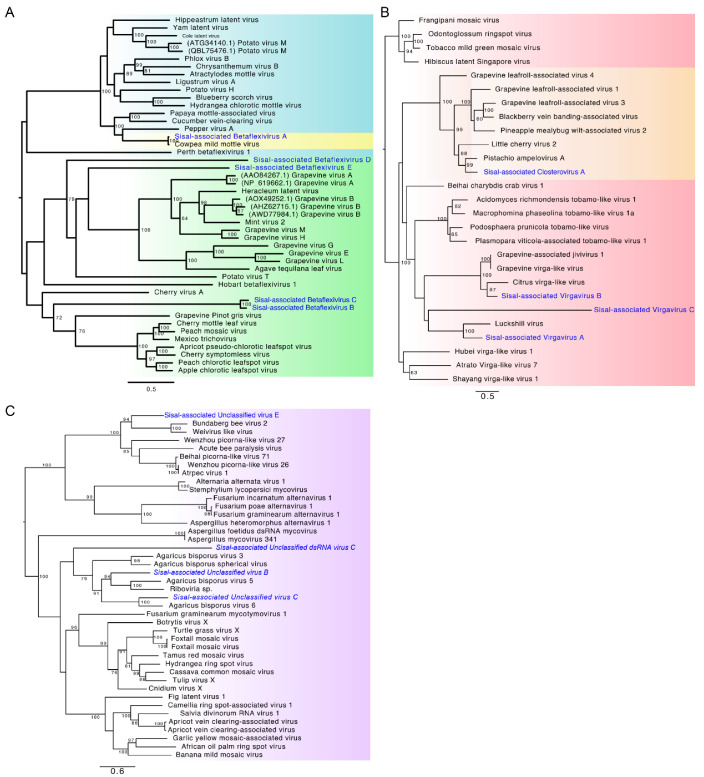
Phylogenetic analysis of high-confidence viral sequences identified in *Agave* species. Phylogenetic trees contain 13 selected species, highlighted in blue. (**A**) Family Betaflexiviridae. In blue; Carlavirus. In green; Trinivirinae. In yellow; unclassified Betaflexivirus. (**B**) +ssRNA (positive sense, single-stranded RNA), in red. In orange; Ampelovirus. (**C**) Riboviria (in violet). Node support values were determined using 1000 pseudoreplicates where values over 60% of confidence are shown.

In order to further assess the phylogenetic relationship between *Agave*-associated viruses and related species in public reference databases, we selected all the species belonging to Classes 2–4 to perform phylogeny, which included 13 putative viral species (Figure 3A–C). Among these 13 species, five were assigned to the family *Betaflexiviridae* (Figure 3A): *Sisal-associated Betaflexivirus A* (phylogenetically closer to *Cowpea mild mottle virus*, a broad-host-range plant-infecting species also found in our samples); *Sisal-associated Betaflexivirus B* and *Sisal-associated Betaflexivirus C* formed a separate clade, phylogenetically closer to a clade including the *Grapevine Pinot gris virus* and others, all of which characterized as plant-infecting species; *Sisal-associated Betaflexivirus E* was closer to a clade including the *Agave tequilana leaf virus* (virus identified in a species related to the *Agave* species investigated in our study)*, Heracleum latent virus*, and seven other viral species infecting grapevine. Three viral species were assigned to the family Virgaviridae (Figure 3B): *Sisal-associated virgavirus A* was closer to *Luckshill virus*, a virus found in *Drosophila suzukii*, according to Medd et al. (2018) [49]; *Sisal-associated Virgavirus B* (closer to two grapevine viruses), and *Sisal-associated Virgavirus C* (closer to oomycete-infecting viruses and mycoviruses). Three species were designed to clades containing viruses described solely as ‘Unclassified’, besides one *Unclassified dsRNA virus* (Figure 3C): *Sisal-associated Unclassified virus B*, *Sisal-associated Unclassified virus C*, *Sisal-associated Unclassified virus E*, and *Sisal-associated Unclassified dsRNA virus C*. The family *Closteroviridae* were represented by one species (Figure 3B): *Sisal-associated Closterovirus A*. Usually, viral species were assigned to the same family of its closest related virus identified by sequence similarity searches, except for *Sisal-associated Virgavirus B*, which was initially described as unclassified, but further phylogenetic analysis indicates it is an element from the Virgaviridae family, similarly to *Sisal-associated Unclassified dsRNA virus*, initially only classified as *Riboviria*. After performing the phylogeny, based on phylogenetic relationships and sequence similarity results we settled the identity and named our 24 putative new viral species, as described in Table 1. We also constructed a phylogenetic tree to analyze the obtained CPMMV isolate, which is available in Figure S4. Interesting, we observed the CPMMV identified in *Agave* species is closely related to other isolates identified in Brazilian samples. Furthermore, we observed a great number of CPMMV isolates derived from different species, indicating the broad range of hosts that the virus can infect. The accession codes for other viral sequences are available in Table S8.

### 3.5. Viral Diversity in Agave Species

**Figure 4:**
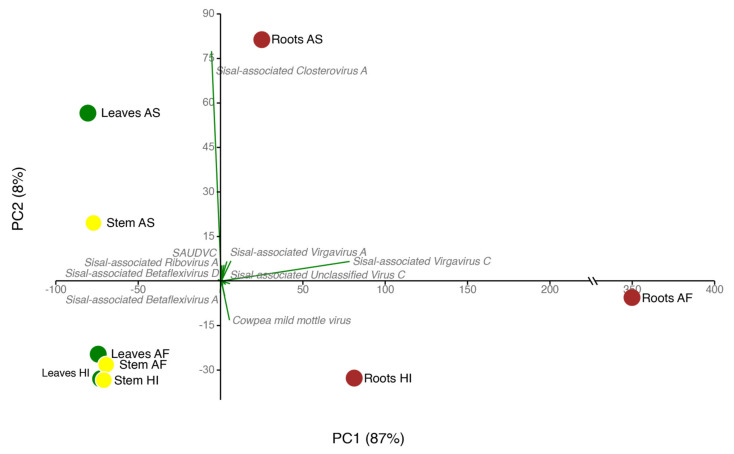
Principal component analysis of virus abundance in *Agave* organs. Viral species were used according to their abundance (green arrows) and presence in organs; Each organ (dots) is represented by a color: leaves (green dots), stems (yellow dots), and roots (brown dots).

In order to further investigate and better understand the viral diversity in our samples, we performed diverse statistical tests (Appendix A). First, we analyzed data normality with Shapiro–Wilk tests and assessed statistical differences among organs with the Kruskal–Wallis test, considering both individual differences in viral diversity and whole viral composition considering viral abundance. Our results (available on Supplementary Materials) indicate that there is no significant difference when comparing the same organs (leaves, stems, roots) among the three sisal taxa. Nonetheless, when comparing the diversity of the organs in the same plant taxa, different patterns could be observed. In *Agave fourcroydes,* the roots displayed a significant difference from the leaves (Kruskal–Wallis test *p*-value 0.003377) and stems (Kruskal–Wallis test *p*-value 0.003723), while in *Agave* Hybrid 11648 the leaves were statistically different from the other organs (Kruskal-Wallis test *p*-values 0.03518 and 0.006339, when comparing against stems and roots, respectively). In *Agave sisalana,* no statistical differences were detected among distinct organs. Considering these results, we decided to portray the results at the organ level, instead of the plant taxa level. Our gamma diversity was 25 viral species, divided with an average alpha species richness of 10.66 species per sample (Table S9). The entropy (Shannon diversity index) in our samples varied greatly. Low values, such as seen in the leaves and stems of *A. sisalana*, and the leaves of *Agave* Hybrid 11648, indicated low richness (in the leaves) or a high dominance of a single species, in this case, the *Sisal-associated Closterovirus A.* The average dominance was lower in the roots, which exhibited a higher richness of species more equally distributed, represented here by the values of evenness (a value based on how similar are the abundance of each species in a sample and the equitability (Simpson’s Diversity Index, a value between 0 and 1 based on the richness and relative abundances in a sample). These values, followed by high dominance, were higher in the leaves and stems of *A. sisalana.* Our principal component analysis (PCA, Figure 4) allowed us to assess the divergence among viral profiles for the three distinct organs analyzed for each of the three *Agave* taxa. We noticed that roots showed the highest divergence among samples, followed by *A. sisalana* leaves and stems. On the other hand, all the other samples displayed a similar profile, clustering together (Figure 4). Table S10 shows the raw expression table for each species according to each sample (and the annotated protein domains for each species).

**Figure 5:**
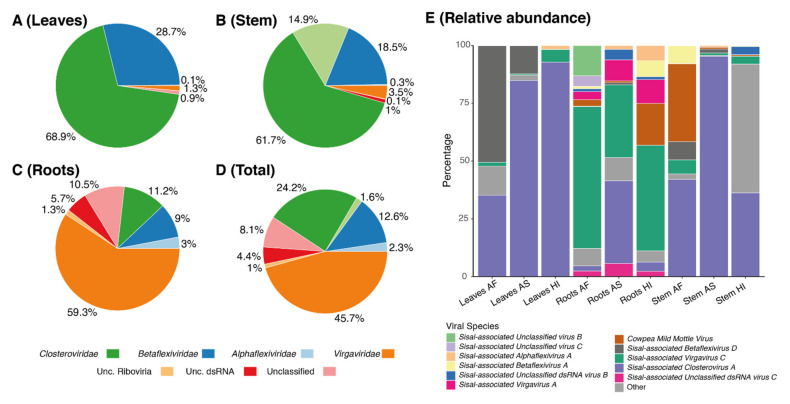
Viral abundance of Agave-associated viruses by organ. Pie charts indicate viral family abundance by expression in tpm, in (**A**): leaves, (**B**): stems, (**C**): roots, and (**D**): in total. Values are summed for all plants. (**E**): The stacked bar plot indicates the relative abundance of the top 12 expressed viral species in each organ.

**Figure 6:**
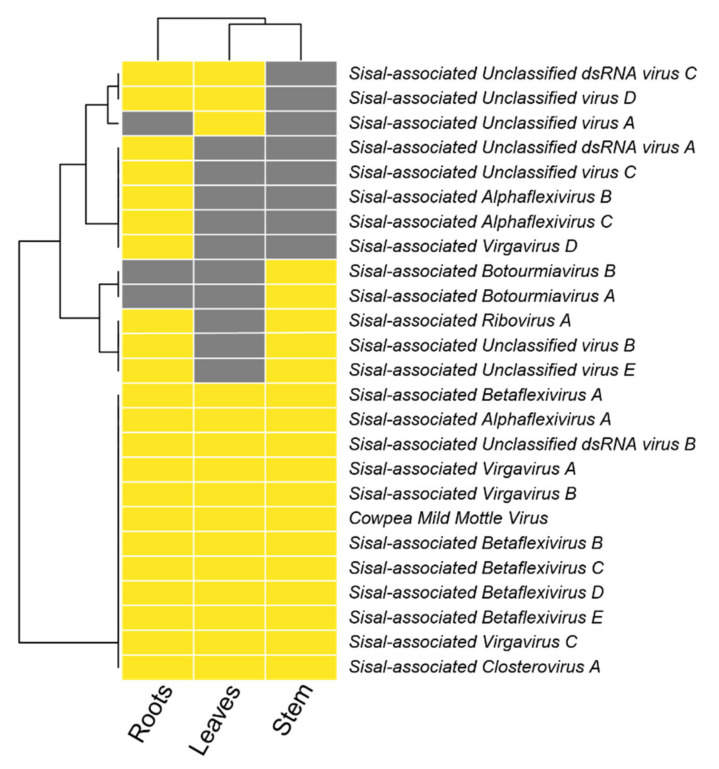
Organ tropism of *Agave*-infecting viruses. Colors indicate the presence (yellow) or absence (grey) of viral species over plant organs.

The richness of viral families varied in all the three organs; however, two viral families represent over 50% of richness in leaves and stems (Figure 5A,B): Betaflexiviridae (represented by five viral species) and Closteroviridae, which was represented by a single species (*Sisal-associated Closterovirus A)*. The roots (Figure 5C) were dominated by viruses from Virgaviridae and exhibited the highest proportion of Unclassified viral species reaching up to 17.5%. We also noted that the roots were the most diverse organ (Figure 6, Table S9), with the number of taxa in all the three plant varieties above the mean *alpha* species richness of 11.66 species per sample. In addition, roots formed a separate clade based on viral species presence/absence (Figure 6), especially due to the *Agave fourcroydes* roots, which possessed the greatest richness of all the organs (19 species) with a low dominance (0.3983).

### 3.6. Organ Tropism of Agave-Infecting Viruses

Abundance assessment of the viral species in each organ showed that a few viral species seem to present systemic infection while others seem to be restricted to a specific organ (Figure 5E). *Sisal-associated Closterovirus A* dominates all leaves and stems, and also the roots of *A. sisalana*, in which it shares dominance with *Sisal-associated Virgavirus C* that has the major contribution in the other root samples, of *A. fourcroydes* and *Agave* Hybrid 11648. The top 12 species with higher transcriptional activity, shown in Figure 5E, represent most of the total abundance in all the organs, while the other 13 species are responsible only for a small portion of viral abundance including the roots of *Agave* Hybrid 11648, where this group of species displays higher abundance than all the other organs. Figure 6 also shows that fewer viral species can be identified in leaves and stems, with only one species exclusively restricted to leaves (*Sisal-associated Unclassified virus A),* two species uniquely found in the stems (*Sisal-associated Botourmiavirus A* and *Sisal-associated Botourmiavirus B*), and other five viral species were restricted to the roots (*Sisal-associated Unclassified dsRNA virus A, Sisal-associated Unclassified virus C, Sisal-associated Virgavirus D, Sisal-associated Alphaflexivirus C*, and *Sisal-associated Alphaflexivirus B*). It is relevant to point out that we observed a high number of putative viruses, 12 species, in co-infection in all three distinct plant organs, which likely indicate they can produce systemic infection.

## 4. Discussion

Plants, such as all the known living organisms, are susceptible to viral infections. Some viral infections are cryptic, i.e., a given virus infects a host with no apparent symptoms [50,51]. In this study, we have described 25 cryptical viral species associated with three plant taxa in the genus *Agave*: *Agave fourcroydes*, *Agave sisalana* and *Agave* hybrid 11648 using RNA-sequencing and genomic DNA sequencing for curation. The use of metagenomics-based approaches for the discovery of viral species is seen in research conducted by Charon et al. [52], and Wolf et al. [53], and also reviewed by Greninger [54], Maclot et al. [55], and Shi et al. [56]. In our methods, we also discarded false-positive results and endogenous elements (EVEs). The plants were grown in a genetic collection, located in an area with very typical climatic and soil conditions of the sisal producing areas in Brazil. To the best of our knowledge, this is the first unbiased virome study in the genus *Agave,* the first virology study in *A. fourcroydes* and A. hybrid 11648, as well as the first virology study in *A. sisalana* since the 1995 study by Izaguirre-mayoral et al. [16], with another two earlier studies on the viral streak of sisal [13,14]. Among the 25 total species described in our study, only one is a known species, *Cowpea mild mottle virus* (CPMMV), and all the other 24 are new viral species. Our approach was based on the similarity of transcripts detected in leaf, stem, and root samples from these three plant taxa to viral nucleotide sequences and protein domains in public databases, followed by the verification of the origin of those sequences via genomic DNA sequencing, removal of false positives, and identity confirmation via phylogeny, when possible.

CPMMV was the only viral species detected in our samples through sequence similarity search at the nucleotide level (Figures S1–S3, Tables S3 and S4). On the other hand, searches at the protein level were able to find sequences (our remaining 24 viral species) sharing lower similarity with known references, thus being considered a new species. CPMMV was first identified by Brunt and Kenten in 1973 [57], infecting the Cowpea (*Vigna unguiculata*, hence the name) in Ghana, and after that in a broad range of other species (*Phaseolus vulgaris, Glycine max, Nicotiana clevelandii, Theobroma cacao*, among others) in vitro. Symptoms included mild to severe mottle chlorosis followed by leaf necrosis, however, visually symptomless individuals, as in our case with the *Agave* species, have been described in this same study. Brunt and Kenten also described how this virus was spread by sap-feeding aphids, however, their results indicate that spreading was dependent on other viruses, such as the *Potato Y virus* or *Pepper Veinal Mottle virus.* Following their discovery, the whitefly (*Bemisia tabacci)* is now widely accepted as the sole vector of CPMMV [58], and the occurrence of the whitefly in the state of Paraíba is also described in the literature [59,60,61], which is suggestive that this insect might also act as the vector for CPMMV in this environment. Nonetheless, CPMMV is more expressed in the stems and roots in all three *Agave* species, not being detected at any levels in the leaves of *A. sisalana* or A. hybrid 11648, and very low levels in the leaves of *A. fourcroydes*, which is in opposition to earlier findings for this species, considering the interaction of its vector (the whitefly) with plant leaves. The isolate PB:AF is also phylogenetically closer to other Brazilian isolates (highlighted in blue), considering all genomes available for this species so far (Figure S4). Of note, CPMMV seems to be a broad range of hosts, since isolates have been identified in many hosts, such as soybean, the common bean, and papaya.

On average, each of our samples contains around 11 species unevenly distributed (Table S9), indicating the presence of dominating species, which we describe in detail in the next paragraphs. In all the three sisal taxa, the roots were the organ with the highest number of species (richness), ranging from 12 species in the root of *A.* Hybrid 11648 and 13 in the roots of *A. sisalana* up to 18 species in the roots of *A. fourcroydes*, the richest sample in our analysis (Table S9). We believe this pattern is due to the diversity of associated microorganisms in the root system [62,63], which are, as the host plant, susceptible to viral infections. Our species have shown similarity with nine mycoviral species at the amino acid level with BLASTx. Indications that these species are also mycoviruses include not only their similarity with known species and phylogeny but also their distribution in plant organs, which is especially higher in roots for such species, making the roots significantly distinct from stem and leaf samples (Figure 5E). The mycoviruses sharing similarities with our species are *Agaricus bisporus virus 5* and *Agaricus bisporus virus 6* [64], *Alternaria alternata virus 1* [65], *Aspergillus foetidus dsRNA mycovirus* [66], *Aspergillus heteromorphus alternavirus 1* [67], *Podosphaera prunicola tobamo-like virus* [68], *Macrophomina phaseolina tobamo-like virus* [69], *Botryosphaeria dothidea tobamo-like virus* (unpublished)*,* and *Stemphylium lycopersici mycovirus* (unpublished) (Table 1). The occurrence of viral species showing similarity with *Aspergillus* mycoviruses, and their pattern of expression (higher in stems and roots, but also present in leaves) leads us to hypothesize that *Aspergillus welwitschiae*, a fungal species which causes the bole rot of sisal [70,71] is also part of the healthy microbiome of sisal, causing disease through imbalances in plant metabolism, rather than infecting vulnerable plants from spores in the environment. Such a pattern has been described in peppermint by Dakin et al. (2010) [72]. Nonetheless, it is also possible that this species is infecting the plant host, and not some associated fungal species, following the theories that mycoviruses originated from plant viruses [73] and that these mycoviruses can replicate in plant cells [74]. We can also hypothesize that such an infection in *A. welwitschiae* could modulate pathogenicity by stimulating it, or causing hypovirulence, as seen in Nuss (2005) [75]. The latter could be a highly promising treatment to the bole rot of sisal if properly managed.

Identification of our likely mycoviral species reveals four species, out of nine, belonging to the family Virgaviridae, the family with the second-highest number of represented contigs (Figure 5E). This viral family is commonly described as only infecting plants [76]; however, the first mycovirus belonging to this family was described by [69] and later by Pandey et al. (2018) [68], and, thus, corroborating our findings. As also described by Pandey et al. (2018) [68], *Podosphaera prunicola tobamo-like virus* shows similarity with *Macrophomina phaseolina tobamo-like virus*, such as our proposed new species, *Sisal-associated Virgavirus C*, which is one of the most highly expressed and dominating viral species in our samples (Figure 5E and Figure 6), especially in the roots (Figure 5E), while *Sisal-associated Virgavirus D* shares similarity with *Podosphaera prunicola tobamo-like virus,* unique to the roots. These results reinforce the assertion that the higher species richness in the root system is related to the microbial species associated with sisal varieties. Furthermore, in the family *Virgaviridae, Sisal-associated Virgavirus B* shares similarity with “*Citrus virga-like virus*” [77], which is a plant-infecting species, and is expressed in stems and roots of *A. sisalana* and *A*. hybrid 11648 and leaves of *A. sisalana*). This viral species was, as seen in Matsumura et al. (2017) [77], isolated from the city of Comendador Gomes, in the state of Minas Gerais, Brazil. This location is in the *Cerrado* biome, another endangered Brazilian biome that shares similarities with the *Caatinga* biome where our samples came from, including moderately low rainfall and low aboveground biomass [78].

Besides the aforementioned *Citrus virga-like virus* and *Cowpea Mild Mottle virus*, the other 12 species sharing similarity with our discoveries are plant-infecting viruses. The most prominent of those species, as seen in Figure 5E, is *Sisal-associated Closterovirus A,* the only representative species of the family *Closteroviridae*, which is the most represented viral family in our samples, sharing similarity with *Pistachio ampelovirus A*, first described by Al Rwahnih et al. (2018) [79]. This species is present in all the samples and plant taxa (Figure 5E and Figure 6) but is especially highly expressed in *A. sisalana* (Figure 5E). This +ssRNA family has been described by Rubio et al. (2013) [80] as transmitted through mealybugs, aphids, or the whitefly, which is also likely responsible for the presence of CPMMV in our samples. The occurrence of mealybugs is described by da Silva et al. in the state of Paraíba, affecting cultivation of cotton (2013) [81] and peanuts (2018) [82], reinforcing the role of this insect in the transmission of viral infections in this region. Our results also revealed six viral species sharing similarities with viruses initially described in grapevines, three of which belonging to the family *Betaflexiviridae* (the third most represented family in the stems, and fourth most represented in leaves and roots); *Sisal-associated Betaflexivirus C*, sharing similarity with *Grapevine Pinot gris virus*, as seen in Giampetruzzi et al. (2012) [51] and *Sisal-associated Betaflexivirus E*, sharing similarity with *Grapevine virus H*, as seen in Candresse et al. (2018) [50], both of which are unique to *A. fourcroydes,* with higher expression in the leaves. *Sisal-associated Betaflexivirus D*, sharing similarity with *Grapevine virus G* as seen in Blouin et al. (2018) [83], which was absent in the organs of A. hybrid 11648, and *Sisal-associated Ribovirus A*, sharing similarity with *Grapevine virga-like virus* (unpublished), which is unique to the stems and roots of *A. sisalana* and A. hybrid 11648. The family Betaflexiviridae, which also includes CMMV, affects exclusively plants [84], and also includes the Sisal-*associated Betaflexivirus A* (sharing similarity with *Apple stem pitting virus*), not expressed in *A. sisalana,* and *Sisal-associated Betaflexivirus B* (sharing similarity with *Diuris virus A*), not expressed in *A.* hybrid 11648. Taking together, our findings indicate that there is no organ tropism for the family Betaflexiviridae in our samples, even though some species seem to favor the leaves or roots. Other two species (*Sisal-associated Botourmiavirus A* and *Sisal-associated Botourmiavirus B*) share similarities with the oomycete-infecting viral species, *Plasmopara viticola associated ourmia-like virus 29* and *Plasmopara viticola associated ourmia-like virus 6*, respectively, both of which were described by Chiapello et al. (2020) [85]. Since the production of grapes in the state of Paraíba [86] and the *caatinga* biome [87] is limited, with a local study by Medeiros et al. (2017) [88] considering the climate conditions unsuitable for the cultivation of vines, the similarity of some of our new viral species to grapevine-infecting viruses suggests that these species have a broader host range than just the species from which they were originally isolated.

The family Alphaflexiviridae includes three species in our samples, sharing similarities on BLASTx with plant-infecting species in this same family; *Alternanthera mosaic virus* [89], *Cassia mild mosaic virus* [90], and *Nerine virus X* [91]. As well as Betaflexiviridae, this family does not display an expression pattern to the family level. *Sisal-associated Alphaflexivirus A* is expressed only in the stems and roots of *A. sisalana* and the hybrid, and in the leaves of *A. sisalana* while *Sisal-associated Alphaflexivirus B* is exclusive of the roots of *A. fourcroydes* and *A. sisalana*, and *Sisal-associated Alphaflexivirus C* is unique to the roots of *A. fourcroydes. Sisal-associated Unclassified virus A* is also unique to *A. fourcroydes* but to leaves instead of roots.

Finally, a curious result is the detection of *Sisal-associated Unclassified virus E*, which shares similarity on BLASTx with *Halhan virus 3*, described by Rosani et al. (2019) [92]. This viral species was infecting the bivalve *Haliotis discus*, a sea snail species. By contrast with the aforementioned fungal and plant species, the occurrence of sea snails in the Brazilian *caatinga* is virtually impossible. Thus, considering the expression of *Sisal-associated Unclassified virus E* only in the stems and roots of *A. fourcroydes,* we hypothesize that other *Gastropoda* species inhabiting the roots of this plant, as described by Pearce and Örstan (2006) [93] and Pratt (1971) [94], might have left viable viral RNA on the organs where it was detected, and that this group of species occurs in a broader range of environments than previously thought.

## 5. Conclusions

This is the first study using an unbiased high-throughput sequencing strategy to investigate the viral diversity in the genus *Agave*. According to our initial hypothesis that *Agave* species could act as a reservoir of plant viruses even with no apparent infection, in our study we were able to identify 25 species associated with three sisal taxa, of which 24 likely represent new viral species. Most of the species displayed high transcriptional activity in the roots, the plant organ with the highest viral diversity. At the species level, *Agave fourcroydes* is the variety with a higher abundance of viral species. A total of 11 new viral species shares similarities with known mycoviruses and oomycete-infecting viral species, reinforcing the effects of the associated microbiota in viral diversity.

## Figures and Tables

**Table 1:** Viral species assignments according to sequence similarity searches and phylogeny. Columns indicate (i) the family indicated by phylogeny, (ii) the family of the best BLASTx hit (columns merged when results are identical), (iii) the species of the best BLASTx hit, and (iv) the new species name.

Family (BLAST)	Family (Phy)	Species Hit (BLASTx)	New Name
Unclassified	-	Actinidia seed-borne latent virus	Sisal-associated Unclassified virus A
	Unclassified	Agaricus bisporus virus 5	Sisal-associated Unclassified virus B
	Unclassified	Agaricus bisporus virus 6	Sisal-associated Unclassified virus C
Alphaflexiviridae	-	Alternanthera mosaic virus	Sisal-associated Alphaflexivirus A
Unclassified dsRNA	-	Alternaria alternata virus 1	Sisal-associated Unclassified dsRNA virus A
	Betaflexiviridae	Apple stem pitting virus	Sisal-associated Betaflexivirus A
Unclassified dsRNA	-	Aspergillus foetidus dsRNA mycovirus	Sisal-associated Unclassified dsRNA virus B
Unclassified	-	Aspergillus heteromorphus alternavirus 1	Sisal-associated Unclassified virus D
	Virgaviridae	Botryosphaeria dothidea tobamo-like virus	Sisal-associated Virgavirus A
Alphaflexiviridae	-	Cassia mild mosaic virus	Sisal-associated Alphaflexivirus B
Unclassified	Virgaviridae	Citrus virga-like virus	Sisal-associated Virgavirus B
	Betaflexiviridae	Diuris virus A	Sisal-associated Betaflexivirus B
	Betaflexiviridae	Grapevine Pinot gris virus	Sisal-associated Betaflexivirus C
Unclassified Riboviria	-	Grapevine virga-like virus	Sisal-associated Ribovirus A
	Betaflexiviridae	Grapevine virus G	Sisal-associated Betaflexivirus D
	Betaflexiviridae	Grapevine virus H	Sisal-associated Betaflexivirus E
	Unclassified	Halhan virus 3	Sisal-associated Unclassified virus E
	Virgaviridae	Macrophomina phaseolina tobamo-like virus	Sisal-associated Virgavirus C
Alphaflexiviridae	-	Nerine virus X	Sisal-associated Alphaflexivirus C
	Closteroviridae	Pistachio ampelovirus A	Sisal-associated Closterovirus A
Botourmiaviridae	-	Plasmopara viticola associated ourmia-like virus 29	Sisal-associated Botourmiavirus A
Botourmiaviridae	-	Plasmopara viticola associated ourmia-like virus 6	Sisal-associated Botourmiavirus B
Virgaviridae	-	Podosphaera prunicola tobamo-like virus	Sisal-associated Virgavirus D
Unclassified Riboviria	Unclassified dsRNA	Stemphylium lycopersici mycovirus	Sisal-associated Unclassified dsRNA virus C

## Data Availability

The data supporting the reported results in this research can be found under the accession code *PRJNA746623* in SRA and under accession codes MZ329754-MZ329767 and MZ599598-MZ599651 in the Nucleotide Database of NCBI.

## Supplementary Materials

The following are available online at https://www.mdpi.com/article/10.3390/microorganisms9081704/s1, Table S1: Species names and respective accession numbers of the viral sequences used for phylogenetic analysis in Figure 3; Table S2: General description of deep sequenced libraries and virus detection strategy. Raw counts of RNA and DNA libraries as well as metrics related to transcript assembly and sequence similarity searches are presented in the rows. *Agave* species are indicated in the columns; Table S3: Similarity of *Agave*-derived contigs against known CPMMV genomes. Analysis was performed using Blast with the variant BLASTn; Table S4: Quantitative metrics for the reference genome of CPMMV (CPMMV:BR:MG:09:3) and the genome obtained from the RNA-seq data of *Agave fourcroydes*; Table S5: BLASTx best hits for each viral contig, and their respective alignment metrics; Table S6: Transcripts discarded as false positives (rows) and associated data. Columns indicate transcript name, length (in base pairs), the protein and species hit on BLASTx, their respective accession code, detected protein domains, and finally, the reason for discard; Table S7: A classification scheme for phylogeny. Columns indicate: Class name, the requisites for that given class (characteristics of the viral species, concerning contig length and type), the number of species included in that category, and their correspondent percentage; Figure S1: Alignment of detected transcripts (arrows in the lower portion) of the three plant varieties sharing similarity with the genome of *Cowpea Mild Mottle virus* (KC884245.1), in the upper half of the figure, using IGV. Colorful lines inside arrows indicate genetic variation in comparison with the reference; Figure S2: Raw sequencing reads (with length >50 bp) coverage of the reference genome of CPMMV using IGV. Each arrow in the lower part indicates a single sequencing read. In those arrows, colorful regions indicate genetic variation in comparison to the reference. As seen in the middle section, coverage is deeper in the end on the transcript; Figure S3: ORF pattern in CPMMV:BR:MG:09:3 (upper) and CPMMV isolate PB:AF (lower). Figure S4: Phylogenetic analysis of Cowpea Mild Mottle virus isolate PB:AF. Accession numbers in blue indicate samples from Brazil, while green indicates the isolate assembled in this work; Table S8: The accession codes and descriptions to each viral contig and their respective viral species. “RdRp” stands for RNA-dependent RNA polymerase, and, in this table, is a synonym to “Replicase”; Table S9: Diversity indices for the organs and species analyzed; Table S10: Viral species and their respective detected protein domains (with Pfam/HMMER) and their expression (in tpm, *transcripts per million*) in each analyzed sample, representing the average expression of the three technical replicates.

## Appendix A

Statistical Analysis among Organs Samples.

Part I: Comparing the same tissue type in the three plant varieties

1. Leaves
  1.1. Normality Tests

**Test**	**Leaves AF**	**Leaves AS**	**Leaves HI**
N	25	25	25
Shapiro–Wilk W	0.842	0.2534	0.2256
p(normal)	3.217 × 10^−9^	2.845 × 10^−10^	1.764 × 10^−10^
p (Monte Carlo)	0.0001	0.0001	0.0001
Conclusion: The three distribution are not Gaussian (normal). We must use a non-parametric test.			

1.2. Kruskal–Wallis Test

H (chi2):	2.25
Hc (tie corrected):	3.477
p (same):	0.1758
Conclusion: There is no significant difference between sample medians.	

2. Stem
  2.1. Normality Tests

**Test**	**Stem AF**	**Stem AS**	**Stem HI**
N	25	25	25
Shapiro–Wilk W	0.438	0.2153	0.4168
p(normal)	9.652 × 10^−9^	1.484 × 10^−10^	6.209 × 10^−9^
p (Monte Carlo)	0.0001	0.0001	0.0001
Conclusion: the three distribution are not Gaussian (normal). We must use a non-parametric test.			

2.2. Kruskal–Wallis Test

H (chi2):	0.2957
Hc (tie corrected):	0.3705
p (same):	0.8309
Conclusion: There is no significant difference between sample medians.	

3. Roots
  3.1. Normality Tests

**Test**	**Roots AF**	**Roots AS**	**Roots HI**
N	25	25	25
Shapiro–Wilk W	0.3351	0.4847	0.4686
p(normal)	1.246 × 10^−9^	2.65 × 10^−8^	1.858 × 10^−8^
p (Monte Carlo)	0.0001	0.0002	0.0001
Conclusion: the three distribution are not Gaussian (normal). We must use a non-parametric test.			

3.2. Kruskal–Wallis Test

H (chi2):	2.254
Hc (tie corrected):	2.443
p (same):	0.2948
Conclusion: There is no significant difference between sample medians.	

Part II: Comparing all tissue in of a same plant variety

4. *Agave fourcroydes*
  4.1. Normality Tests

**Test**	**Leaves**	**Stem**	**Roots**
N	25	25	25
Shapiro-Wilk W	0.842	0.438	0.3351
p(normal)	3.217 × 10^−9^	9.652 × 10^−9^	1.246 × 10^−9^
p (Monte Carlo)	0.0001	0.0002	0.0001
Conclusion: the three distribution are not Gaussian (normal). We must use a non-parametric test.			

4.2. Kruskal–Wallis Test

H (chi2):	10.52
Hc (tie corrected):	11.96
p (same):	0.002533
Conclusion: There is a significant difference on viral diversity among distinct tissues in AF.	

4.3. Mann–Whitney Pairwise Test

**Test**	**Leaves**	**Stem**	**Roots**
Leaves		0.9041	0.003377
Stem	0.9041		0.003723
Roots	0.003377	0.003723	
Conclusion: Viral diversity of the roots is statistically significant different from both the diversity in leaf and stem, but there is no significant difference between leaf and stem viral diversity.			

5. *Agave sisalana*
  5.1. Normality Tests

**Test**	**Leaves**	**Stem**	**Roots**
N	25	25	25
Shapiro–Wilk W	0.2534	0.2153	0.4847
p(normal)	2.85 × 10^−7^	1.48 × 10^−7^	2.65 × 10^−8^
p (Monte Carlo)	0.0001	0.0001	0.0001
Conclusion: the three distribution are not Gaussian (normal). We must use a non-parametric test.			

5.2. Kruskal–Wallis Test

H (chi2):	4.006
Hc (tie corrected):	5.019
p (same):	0.0813
Conclusion: There is no significant difference on viral diversity among the three distinct tissues in *Agave sisalana*.	

6. Agave Hybrid 11648
  6.1. Normality Tests

**Test**	**Leaves**	**Stem**	**Roots**
N	25	25	25
Shapiro–Wilk W	0.2256	0.4168	0.4686
p(normal)	1.76 × 10^−7^	6.21 × 10^−6^	1.86 × 10^−5^
p (Monte Carlo)	0.0001	0.0002	0.0001
Conclusion: the three distribution are not Gaussian (normal). We must use a non-parametric test.			

6.2. Kruskal–Wallis Test

H (chi2):	6.226
Hc (tie corrected):	8.437
p (same):	0.01472
Conclusion: There is a significant difference between sample medians.	

6.3. Mann–Whitney Pairwise Test

**Test**	**Leaves**	**Stem**	**Roots**
Leaves		0.03518	0.006339
Stem	0.03518		0.2202
Roots	0.006339	0.2202	
Conclusion: The viral diversity in the leaf is statistically significant different from both stem and roots, but there is no significant difference between the viral diversities of stem and roots.			
