# Supplementary material for: The Sisal Virome: Uncovering the Viral Diversity of Agave Varieties Reveals New and Organ-Specific Viruses
Source: Microorganisms. 2021 Aug 10;9(8):1704. doi: 10.3390/microorganisms9081704 (PMC8400513; doi:10.3390/microorganisms9081704)
Supplement: Supplementary file 1 [file microorganisms-09-01704-s001.zip › microorganisms-1263423-supplementary.pdf]

**Supplementary Data**

**Table S1:** Species names and respective accession numbers of the viral sequences used for phylogenetic analysis in Fig. 3.

| Virus name | Accession |
|---|---|
| *Acidomyces richmondensis* tobamo-like virus 1 | AZT88673 |
| Acute bee paralysis virus | QGL51678 |
| African oil palm ring spot virus | YP_002776347 |
| *Agaricus bisporus* spherical virus | AQM32769 |
| *Agaricus bisporus* virus 3 | AQM49919 |
| *Agaricus bisporus* virus 5 | AQM49927 |
| *Agaricus bisporus* vírus 6 | AQM49948 |
| *Agave tequilana* leaf virus | YP_009373228 |
| *Alternaria alternata* vírus 1 | YP_001976150 |
| Apple chlorotic leaf spot virus | AOF42977 |
| Apple chlorotic leaf spot virus | NP_040551 |
| Apple stem grooving virus | NP_044335 |
| Apple stem pitting virus | NP_604464 |
| Apricot pseudo-chlorotic leaf spot virus | BCA25706 |
| Apricot vein clearing associated virus | YP_008997790 |
| Apricot vein clearing associated virus | BCA25721 |
| *Aspergillus foetidus* dsRNA mycovirus | YP_007353985 |
| *Aspergillus heteromorphus* alternavirus 1 | AZT88576 |
| *Aspergillus* mycovirus 341 | ABX79997 |
| Atractylodes mottle virus | YP_009508317 |
| Atrato Virga-like virus 7 | QHA33778 |
| Atrpec virus 1 | AYN75539 |
| *Auricularia heimuer* myco virgavirus 1 | QIM57886 |
| Banana mild mosaic virus | NP_112029 |
| Beihai charybdis crab vírus 1 | YP_009333242 |
| Beihai picorna-like vírus 71 | YP_009333456 |
| Betavulgaris mito virus 1 | QBN22178 |
| Blackberry vein banding-associated virus | YP_008411010 |
| Blueberry scorch virus | AAY18409 |
| *Botrytis* virus X | NP_932306 |
| Bundaberg bee vírus 2 | AWK77852 |
| Camellia ring spot associated vírus 1 | QEJ80622 |
| *Cannabis sativa* mitovirus 1 | DAB417542 |
| Carrot Ch virus 1 | NP_009103999 |
| Cassava common mosaic virus | NP_042695 |
| *Chenopodium quinoa* mitovirus 1 | YP_009551903 |
| Cherry mottle leaf virus | AOY07776 |
| Cherry rusty mottle associated virus | YP_007761581 |
| Cherry symptomless virus | QIA61761 |
| Cherry virus A | ATJ05013 |
| Chrysanthemum virus B | CAO78688 |
| Citrus chlorotic leaf spot virus | QOQ52500 |
| Citrus leaf blotch virus | NP_624333 |
| Citrus virga-like virus | ARO38275 |
| Cnidium virus X | BBI37360 |
| Cole latent virus | QGN03507 |
| Cowpea mild mottle virus | AGS13088 |

| | |
|---|---|
| Cucumber vein-clearing virus | YP_009664734 |
| Diuris virus A | YP_006905850 |
| Fig latent virus 1 | CAY32622 |
| Fox tail mosaic virus | AWT40556 |
| Fox tail mosaic virus | ABW25054 |
| Frangipani mosaic virus | AEW67306 |
| *Fusarium graminearum* alternavirus 1 | YP_009449446 |
| *Fusarium graminearum* mycotymo virus 1 | YP_009553357 |
| *Fusarium incarnatum* alternavirus 1 | AYJ09266 |
| *Fusarium poae* alternavirus 1 | YP_009272949 |
| Garlic yellow mosaic-associated virus | AZM69107 |
| Grapevine associated jivivirus 1 | QIJ25699 |
| Grapevine leafroll-associated vírus 1 | QBZ78645 |
| Grapevine leafroll-associated vírus 3 | AXI82159 |
| Grapevine leafroll-associated vírus 4 | ARP51817 |
| Grapevine Pinotgris virus | QEQ50023 |
| Grapevine virga-like virus | QCF47402 |
| Grapevine virus A | NP_619662 |
| Grapevine vírus A | AAO84267 |
| Grapevine virus B | AHZ62715 |
| Grapevine virus B | AWD77984 |
| Grapevine vírus B | AOX49252 |
| Grapevine virus E | YP_002117775 |
| Grapevine virus G | ATV81261 |
| Grapevine vírus G | YP_009551946 |
| Grapevine vírus H | YP_009551905 |
| Grapevine vírus L | QBM91193 |
| Grapevine vírus M | QCF24338 |
| *Haemonchus contortus* virus | CDJ82925 |
| Heracleum latent virus | QIQ28218 |
| Hibiscus latent Singapore virus | ARM71140 |
| Hippeastrum latente virus | YP_002308447 |
| Hobart betaflexivirus 1 | AWK77906 |
| Hubei virga-like vírus 1 | YP_009337423 |
| *Humulus lupulus* mitovirus 1 | DAB41749 |
| Hydrangea chlorotic mottle virus | YP_002985636 |
| Hydrangea ring spot virus | BAU45634 |
| Ligustrum vírus A | YP_009288956 |
| Little cherry vírus 2 | ATB18128 |
| Luckshill virus | AWA82251 |
| *Macrophomina phaseolina* tobamo-like virus 1A | ALD891032 |
| Mexico tricho virus | QDR50348 |
| Mint virus 2 | YP_009664761 |
| Odonto glossum rings potvirus | AAZ81884 |
| *Oxybasis rubra* mitovirus 1 | DAB41745 |
| Papaya mottle associated virus | QIJ97108 |
| Peach chlorotic leaf spot virus | AYA62500 |
| Peach mosaic virus | YP_002308565 |
| Pepper virus A | YP_009357230 |
| Perth betaflexivírus 1 | AWK77908 |
| *Petunia exserta* mitovirus 1 | DAB41750 |
| Phlox virus B | YP_001552317 |
| Pineapple mealy bug wilt-associated virus 2 | QCC20262 |
| Pistachio ampelo virus A | AVN99305 |
| *Plasmopara viticola* associated tobamo-like virus 1 | QIP68002 |
| *Podosphaera prunicola* tobamo-like virus | ATS94407 |

| | |
|---|---|
| Potato vírus H | AYV96574 |
| Potato virus M | QBL75476 |
| Potato virus M | ATG34140 |
| Potato virus M | NP_056767 |
| Potato virus T | AFV39891 |
| Potato virus T | YP_002019748 |
| Potato vírus T | AFV39891 |
| Riboviria sp | QDH89210 |
| *Salvia divinorum* RNA virus 1 | YP_009553026 |
| Shayang virga-like vírus 1 | YP_009333208 |
| *Solanum chacoense* mitovirus 1 | DAB41743 |
| *Stemphylium lycopersici* mycovirus | YP_009551661 |
| Tamus red mosaic virus | YP_004849314 |
| Tobacco mild green mosaic virus | QIM41095 |
| Tulip virus X | NP_702988 |
| Turtle grass vírus X | YP_009552762 |
| Weivirus-like virus | QJI53767 |
| Wenzhou picorna-like vírus 26 | YP_009337682 |
| Wenzhou picorna-like vírus 27 | YP_009336706 |
| Yam latente virus | YP_009134730 |

**Table S2:** General description of deep sequenced libraries and virus detection strategy. Raw counts of RNA and DNA libraries as well as metrics related to transcript assembly and sequence similarity searches are presented in the rows. *Agave* species are indicated in the columns.

| | ***A. fourcroydes*** | ***A. sisalana*** | **A. hybrid 11648** | **Sum** |
|---|---|---|---|---|
| **Raw RNAseq reads** | 200,498,222 | 208,260,315 | 150,509,074 | 559,267,611 |
| **Assembled Transcripts** | 81,190 | 93,569 | 77,194 | 251,953 |
| **Mean contig length** | 599 | 575 | 584 | 1758 |
| **Min. contig length** | 168 | 171 | 171 | 510 |
| **Viral Transcripts BLASTN** | 4 | 1 | 5 | 10 |
| **Viral Transcripts BLASTX** | 49 | 34 | 28 | 79* |
| **Raw DNAseq reads** | 131,365,537 | 118,820,218 | 119,180,610 | 369,366,365 |

**Table S3:** Similarity of *Agave*-derived contigs against known CPMMV genomes. Analysis was performed using Blast with the variant BLASTn.

| | **Plant** | AF | | | | AS | HI | | | | |
|---|---|---|---|---|---|---|---|---|---|---|---|
| | **Contig** | DN22786 | DN53839 | DN59783 | DN97584 | DN6612 | DN10130 | DN110105 | DN114005 | DN42686 | DN55516 |
| | **Length** | 270 | 3600 | 369 | 362 | 867 | 867 | 264 | 387 | 315 | 285 |
| **KC774019.1** | **Sim. (%)** | 97.41 | 98.31 | 98.37 | 98.34 | 98.62 | 98.73 | 98.49 | 95.87 | 98.41 | 98.25 |
| | **Length** | 269 | 3599 | 368 | 361 | 866 | 866 | 263 | 386 | 314 | 284 |
| **KC774020.1** | **Sim. (%)** | 97.78 | 98.08 | 97.29 | 97.21 | 98.85 | 98.96 | 98.86 | 95.35 | 98.73 | 97.54 |
| | **Length** | 269 | 3599 | 368 | 358 | 866 | 866 | 263 | 386 | 314 | 284 |
| **KC884245.1** | **Sim. (%)** | 97.05 | 98.42 | 98.37 | 98.62 | 98.85 | 98.96 | 99.24 | 95.87 | 97.46 | 96.49 |
| | **Length** | 270 | 3599 | 368 | 361 | 866 | 866 | 263 | 386 | 314 | 284 |
| **MK202583.1** | **Sim. (%)** | 98.52 | 96.25 | 98.37 | 96.41 | 90.31 | 90.43 | 99.24 | 94.32 | 96.19 | |
| | **Length** | 269 | 3595 | 368 | 361 | 866 | 866 | 263 | 386 | 314 | |
| **KC884248.1** | **Sim. (%)** | 98.15 | 98.28 | 98.65 | 98.34 | | | 99.24 | 95.87 | 97.46 | |
| | **Length** | 269 | 3599 | 368 | 361 | | | 263 | 386 | 314 | |

**Table S4:** Quantitative metrics for the reference genome of CPMMV (CPMMV:BR:MG:09:3) and the genome obtained from the RNA-seq data of *Agave fourcroydes.*

| Name | CPMMV:BR:MG:09:3 | CPMMV isolate PB:AF |
|---|---|---|
| **Contigs** | 1 | 1 |
| **Length** | 8196 | 8193 |
| **N50** | 8196 | 8193 |
| **L50** | 1 | 1 |
| **GC** | 0.403 | 0.4 |
| | **For runs of Ns (>= 10 Ns):** | |
| **Num** | 0 | 2 |
| **Span** | 0 | 47 |

**Table S5:** BLASTx best hits for each viral contig, and their respective alignment metrics.

| qseqid (TRINITY_DN...) | sseqid | pident | length | mismatch | gapopen | qstart | qend | sstart | send | evalue | bitscore | qlen | species |
|---|---|---|---|---|---|---|---|---|---|---|---|---|---|
| 61147 | YP_009553503.1 | 50.9 | 55 | 27 | 0 | 4 | 168 | 1915 | 1969 | 8.10E-14 | 65.9 | 213 | Actinidia seed-borne latent virus |
| 49676 | AQM49927.1 | 39.7 | 662 | 384 | 7 | 4 | 1953 | 1742 | 2400 | 5.2E-124 | 435.3 | 2163 | Agaricus bisporus virus 5 |
| 16142 | AQM49927.1 | 32.7 | 431 | 252 | 8 | 115 | 1356 | 1259 | 1668 | 1.90E-60 | 223.4 | 1359 | Agaricus bisporus virus 5 |
| 44352 | AQM49927.1 | 30.8 | 347 | 226 | 6 | 4 | 1008 | 1742 | 2086 | 2.20E-37 | 146.4 | 1011 | Agaricus bisporus virus 5 |
| 83710 | AQM49927.1 | 46.3 | 188 | 100 | 1 | 1 | 564 | 2214 | 2400 | 1.40E-42 | 163.3 | 774 | Agaricus bisporus virus 5 |
| 19804 | AQM49927.1 | 52.9 | 140 | 66 | 0 | 7 | 426 | 2076 | 2215 | 4.30E-38 | 147.5 | 435 | Agaricus bisporus virus 5 |
| 24880 | AQM32763.1 | 54.5 | 411 | 187 | 0 | 37 | 1269 | 2132 | 2542 | 1.3E-139 | 486.5 | 1404 | Agaricus bisporus virus 6 |
| 27401 | AQM49948.1 | 47.3 | 281 | 145 | 3 | 1 | 843 | 1815 | 2092 | 2.10E-68 | 249.2 | 846 | Agaricus bisporus virus 6 |
| 77293 | AQM49948.1 | 59.5 | 121 | 47 | 2 | 4 | 366 | 1700 | 1818 | 1.60E-41 | 158.7 | 366 | Agaricus bisporus virus 6 |
| 55189 | AQM32763.1 | 41.1 | 112 | 58 | 3 | 7 | 324 | 642 | 751 | 4.70E-21 | 90.5 | 327 | Agaricus bisporus virus 6 |
| Contig17 | YP_459948.1 | 32.8 | 186 | 121 | 3 | 271 | 825 | 11 | 193 | 1.50E-26 | 110.2 | 867 | Alternanthera mosaic virus |
| 63052 | YP_459944.1 | 43 | 114 | 64 | 1 | 1 | 339 | 1023 | 1136 | 1.40E-20 | 89 | 342 | Alternanthera mosaic virus |
| 81586 | YP_001976142.1 | 85.5 | 110 | 16 | 0 | 1 | 330 | 441 | 550 | 3.10E-52 | 194.1 | 330 | Alternaria alternata virus 1 |
| 61614 | YP_001976151.1 | 75 | 80 | 20 | 0 | 7 | 246 | 21 | 100 | 1.30E-34 | 135.2 | 246 | Alternaria alternata virus 1 |
| Contig7 | NP_604464.1 | 49.9 | 1001 | 477 | 6 | 607 | 3588 | 1193 | 2175 | 1.80E-286 | 975.7 | 3600 | Apple stem pitting virus |
| 83885 | NP_604464.1 | 42.8 | 201 | 112 | 3 | 10 | 606 | 1652 | 1851 | 8.50E-40 | 153.7 | 609 | Apple stem pitting virus |
| Contig14 | YP_007353983.1 | 53.2 | 538 | 250 | 1 | 1 | 1614 | 191 | 726 | 2.10E-170 | 589 | 1617 | Aspergillus foetidus dsRNA mycovirus |

| | | | | | | | | | | | | | |
|---|---|---|---|---|---|---|---|---|---|---|---|---|---|
| 73798 | YP_007353985.1 | 76.3 | 97 | 23 | 0 | 10 | 300 | 637 | 733 | 3.50E-39 | 150.6 | 300 | Aspergillus foetidus dsRNA mycovirus |
| 116043 | YP_007353985.1 | 75.9 | 79 | 19 | 0 | 4 | 240 | 307 | 385 | 1.20E-34 | 135.2 | 240 | Aspergillus foetidus dsRNA mycovirus |
| 72309 | YP_007353983.1 | 55.4 | 56 | 25 | 0 | 64 | 231 | 6 | 61 | 2.60E-13 | 64.3 | 231 | Aspergillus foetidus dsRNA mycovirus |
| Contig19 | AZT88576.1 | 49.6 | 514 | 255 | 3 | 1 | 1530 | 319 | 832 | 9.90E-138 | 480.3 | 1533 | Aspergillus heteromorphus alternavirus 1 |
| 83752 | AZT88575.1 | 55.4 | 112 | 50 | 0 | 1 | 336 | 159 | 270 | 4.70E-40 | 153.7 | 336 | Aspergillus heteromorphus alternavirus 1 |
| Contig5 | QED22727.1 | 54.6 | 1124 | 502 | 5 | 31 | 3384 | 13 | 1134 | 0.00E+00 | 1201.8 | 3387 | Botryosphaeria dothidea tobamo-like virus |
| Contig12 | ADD65542.1 | 41.2 | 148 | 82 | 1 | 1 | 444 | 525 | 667 | 6.50E-32 | 127.1 | 465 | Cassia mild mosaic virus |
| 100760 | ARO38275.1 | 56.7 | 838 | 353 | 3 | 544 | 3036 | 1 | 835 | 4.70E-280 | 954.1 | 3069 | Citrus virga-like virus |
| 26278 | ARO38274.1 | 50.4 | 125 | 61 | 1 | 4 | 378 | 1168 | 1291 | 1.00E-31 | 126.3 | 432 | Citrus virga-like virus |
| 133840 | ARO38274.1 | 62.3 | 114 | 42 | 1 | 1 | 339 | 369 | 482 | 9.20E-36 | 139.4 | 339 | Citrus virga-like virus |
| 88105 | YP_006905850.1 | 32.6 | 218 | 120 | 8 | 1 | 612 | 1107 | 1311 | 1.40E-18 | 83.2 | 615 | Diuris virus A |
| 86319 | YP_006908997.1 | 66.7 | 105 | 28 | 3 | 1 | 309 | 258 | 357 | 5.10E-33 | 130.2 | 309 | Diuris virus A |
| 44239 | YP_004732978.2 | 36.7 | 256 | 139 | 13 | 7 | 744 | 1221 | 1463 | 1.10E-25 | 107.1 | 765 | Grapevine Pinot gris virus |
| 3617 | QCF47403.1 | 38.4 | 739 | 391 | 12 | 70 | 2268 | 25 | 705 | 1.80E-138 | 483.4 | 2310 | Grapevine virga-like virus |
| 65779 | QCF47402.1 | 63.4 | 134 | 49 | 0 | 1 | 402 | 368 | 501 | 1.60E-42 | 162.2 | 402 | Grapevine virga-like virus |
| 52979 | YP_009552539.1 | 26.6 | 872 | 538 | 26 | 31 | 2406 | 16 | 865 | 2.30E-73 | 267.3 | 2619 | Grapevine virus G |
| 46510 | YP_009552539.1 | 25 | 680 | 408 | 23 | 1 | 1806 | 210 | 865 | 8.50E-41 | 158.7 | 1965 | Grapevine virus G |
| 40814 | YP_009552542.1 | 38.3 | 128 | 79 | 0 | 16 | 399 | 74 | 201 | 7.60E-21 | 90.1 | 402 | Grapevine virus G |
| 65618 | YP_009552542.1 | 35.9 | 128 | 82 | 0 | 10 | 393 | 74 | 201 | 2.20E-20 | 88.6 | 396 | Grapevine virus G |

| | | | | | | | | | | | | | |
|---|---|---|---|---|---|---|---|---|---|---|---|---|---|
| 42135 | YP_009551905.1 | 54.9 | 255 | 115 | 0 | 82 | 846 | 1368 | 1622 | 6.20E-81 | 290.8 | 846 | Grapevine virus H |
| 52810 | YP_009552715.1 | 38.4 | 482 | 281 | 7 | 91 | 1521 | 1232 | 1702 | 3.40E-87 | 313.9 | 4224 | Halhan virus 3 |
| 99951 | YP_009552715.1 | 48.6 | 72 | 34 | 3 | 1 | 216 | 445 | 513 | 1.10E-13 | 65.5 | 222 | Halhan virus 3 |
| Contig9 | YP_009109559.1 | 27.4 | 1644 | 930 | 51 | 1093 | 5370 | 2 | 1600 | 3.90E-136 | 476.9 | 5397 | Macrophomina phaseolina tobamo-like virus |
| Contig6 | YP_009109559.1 | 32.9 | 140 | 77 | 4 | 2419 | 2838 | 1314 | 1436 | 4.50E-10 | 57.4 | 3309 | Macrophomina phaseolina tobamo-like virus |
| 30831 | YP_009109561.1 | 41.6 | 370 | 182 | 6 | 1 | 1110 | 40 | 375 | 1.40E-64 | 236.9 | 1110 | Macrophomina phaseolina tobamo-like virus |
| 38090 | YP_009109562.1 | 46.1 | 332 | 169 | 2 | 43 | 1008 | 2 | 333 | 7.30E-84 | 300.8 | 1038 | Macrophomina phaseolina tobamo-like virus |
| Contig8 | YP_009109562.1 | 36.9 | 293 | 178 | 6 | 31 | 888 | 7 | 299 | 3.20E-44 | 169.1 | 1011 | Macrophomina phaseolina tobamo-like virus |
| 105616 | YP_009109562.1 | 36.1 | 299 | 180 | 4 | 16 | 891 | 7 | 301 | 2.20E-45 | 172.9 | 1005 | Macrophomina phaseolina tobamo-like virus |
| 72996 | YP_009109559.1 | 84.3 | 70 | 11 | 0 | 1 | 210 | 1962 | 2031 | 1.60E-33 | 131.3 | 213 | Macrophomina phaseolina tobamo-like virus |
| 26423 | YP_009109559.1 | 48.5 | 68 | 35 | 0 | 1 | 204 | 1766 | 1833 | 3.70E-16 | 73.6 | 204 | Macrophomina phaseolina tobamo-like virus |
| 75281 | YP_009109561.1 | 68.3 | 63 | 20 | 0 | 13 | 201 | 221 | 283 | 3.30E-20 | 87 | 204 | Macrophomina phaseolina tobamo-like virus |
| 85955 | YP_009109559.1 | 84.1 | 63 | 10 | 0 | 7 | 195 | 1351 | 1413 | 2.00E-27 | 110.9 | 198 | Macrophomina phaseolina tobamo-like virus |
| 38752 | YP_446996.1 | 33.8 | 201 | 132 | 1 | 148 | 747 | 16 | 216 | 1.20E-33 | 133.7 | 774 | Nerine virus X |
| 59050 | AVN99304.1 | 41.6 | 1634 | 766 | 18 | 1 | 4899 | 1 | 1446 | 0.00E+00 | 1148.3 | 4902 | Pistachio ampelovirus A |
| 49906 | AVN99304.1 | 42.4 | 1603 | 762 | 17 | 1 | 4794 | 1 | 1446 | 0.00E+00 | 1160.2 | 4797 | Pistachio ampelovirus A |
| Contig16 | AVN99304.1 | 34.8 | 1246 | 655 | 16 | 1 | 3729 | 1 | 1091 | 1.00E-191 | 661 | 3732 | Pistachio ampelovirus A |

| | | | | | | | | | | | | | |
|---|---|---|---|---|---|---|---|---|---|---|---|---|---|
| 59050 | AVN99304.1 | 43.4 | 670 | 378 | 1 | 1 | 2010 | 1 | 669 | 8.20E-159 | 551.6 | 3561 | Pistachio ampelovirus A |
| 2548 | AVN99305.1 | 63.6 | 453 | 165 | 0 | 1 | 1359 | 33 | 485 | 3.30E-177 | 611.3 | 1362 | Pistachio ampelovirus A |
| Contig15 | AVN99306.1 | 39.1 | 207 | 126 | 0 | 292 | 912 | 150 | 356 | 4.10E-38 | 148.7 | 924 | Pistachio ampelovirus A |
| 75741 | QGY72559.1 | 46.6 | 131 | 66 | 3 | 7 | 390 | 459 | 588 | 1.60E-28 | 115.5 | 390 | Plasmopara viticola associated ourmia-like virus 29 |
| 110665 | QGY72536.1 | 61.8 | 68 | 26 | 0 | 1 | 204 | 76 | 143 | 2.30E-21 | 90.9 | 204 | Plasmopara viticola associated ourmia-like virus 6 |
| 83949 | ATS94408.1 | 46.4 | 207 | 107 | 3 | 10 | 621 | 614 | 819 | 2.90E-43 | 165.2 | 621 | Podosphaera prunicola tobamo-like virus |
| 77371 | ATS94409.1 | 40.9 | 171 | 101 | 0 | 61 | 573 | 98 | 268 | 6.80E-39 | 150.6 | 576 | Podosphaera prunicola tobamo-like virus |
| 69322 | ATS94408.1 | 49.4 | 85 | 42 | 1 | 7 | 261 | 821 | 904 | 3.00E-18 | 80.9 | 264 | Podosphaera prunicola tobamo-like virus |
| Contig20 | YP_009551663.1 | 64 | 339 | 114 | 2 | 4 | 1011 | 423 | 756 | 2.90E-125 | 438.3 | 1014 | Stemphylium lycopersici mycovirus |
| Contig1 | YP_009551661.1 | 68.5 | 308 | 87 | 3 | 1 | 924 | 545 | 842 | 8.70E-113 | 396.7 | 927 | Stemphylium lycopersici mycovirus |
| 17344 | YP_009551660.1 | 64.3 | 252 | 90 | 0 | 1 | 756 | 897 | 1148 | 9.70E-102 | 359.8 | 759 | Stemphylium lycopersici mycovirus |
| Contig10 | YP_009551661.1 | 56.5 | 239 | 98 | 1 | 4 | 702 | 305 | 543 | 1.10E-75 | 273.1 | 702 | Stemphylium lycopersici mycovirus |
| Contig11 | YP_009551661.1 | 64.8 | 128 | 45 | 0 | 1 | 384 | 170 | 297 | 1.70E-41 | 158.7 | 387 | Stemphylium lycopersici mycovirus |
| 92324 | YP_009551660.1 | 68 | 100 | 32 | 0 | 1 | 300 | 799 | 898 | 3.30E-40 | 154.1 | 306 | Stemphylium lycopersici mycovirus |

**Table S6:** Overview of the transcripts assigned as false positives (rows) and associated data. Columns indicate transcript name, length (in base pairs), the protein and species hit on BLASTx, their respective accession code, detected protein domains and finally, the reason for discard.

| **Protein Domain** | **Discarded by** | **ORF Pattern** |
|---|---|---|
| AAA domain | Present in genomic DNA, protein domain not viral | non-fragmented |
| Cation efflux family, Dimerisation domain of Zinc Transporter | Present in genomic DNA, protein domain not viral | |
| Thaumatin family | Present in genomic DNA, protein domain not viral | |
| *none identified* | *no relevant hits* | |
| *none identified* | *no relevant hits* | non-fragmented |
| *none identified* | *no relevant hits* | non-fragmented |
| *Cas3* | Protein domain not viral | |
| Viral methyltransferase | CPMMV | |
| *none identified* | Present in genomic DNA | non-fragmented |
| *none identified* | Present in genomic DNA | non-fragmented |
| Mitovirus RdRp | Present in genomic DNA | non-fragmented |
| *none identified* | Present in genomic DNA | non-fragmented |
| *none identified* | Present in genomic DNA | non-fragmented |

| Name | Length | Protein hit | Species | Accesion code |
|---|---|---|---|---|
| *disc1* | 813 | RdRp | Macrophomina phaseolina tobamo-like virus | YP_009109559.1 |
| *disc2* | 1197 | Putative Protein P31 | Pistachio ampelovirus A | AVN99313.1 |
| *disc3* | 762 | Putative Protein P33 | Pistachio ampelovirus A | AVN99309.1 |
| *disc4* | 201 | Hypothetical Protein | Aspergillus foetidus dsRNA mycovirus | YP_007353982.1 |
| *disc5* | 1809 | Putative RdRp | Aspergillus mycovirus 341 | ABX79997.1 |
| *disc6* | 222 | RdRp | Helicobasidium mompa partitivirus V1-2 | BAD32678.1 |
| *disc7* | 792 | Movement Protein | Podosphaera prunicola tobamo-like virus | ATS94408.1 |
| *disc8* | 369 | RdRp | Apple stem pitting virus | NP_604464.1 |
| *disc9* | 693 | RdRp | Beta vulgaris mitovirus 1 | DAB41759.1 |
| *disc10* | 309 | RdRp | Beta vulgaris mitovirus 1 | DAB41759.1 |
| *disc11* | 435 | RdRp | Beta vulgaris mitovirus 1 | DAB41759.1 |
| *disc12* | 207 | RdRp | Beta vulgaris mitovirus 1 | DAB41759.1 |
| *disc13* | 228 | RdRp | Beta vulgaris mitovirus 1 | DAB41759.1 |

**Table S7:** Classification scheme for the phylogenetic analysis. Columns indicate: Class name, the requisites for that given class (characteristics of the viral species, concerning contig length and type), the number of species included in that category, and their correspondent percentage.

| Class | Requisites | Number | (%) |
|---|---|---|---|
| **0** | No RdRp sequence detected | 2 | 9.52 |
| **1** | Partial RdRp sequence (<500 bp) accompanied by other transcripts or not | 10 | 40.00 |
| **2** | Large fragment (>500 bp) of RdRp | 8 | 32.00 |
| **3** | Large fragment (>500 bp) of RdRp and some other viral protein (except for the coat protein) | 1 | 4.00 |
| **4** | Large fragment (>500 bp) of RdRp and the coat protein detected, other viral protein facultative | 4 | 16.00 |

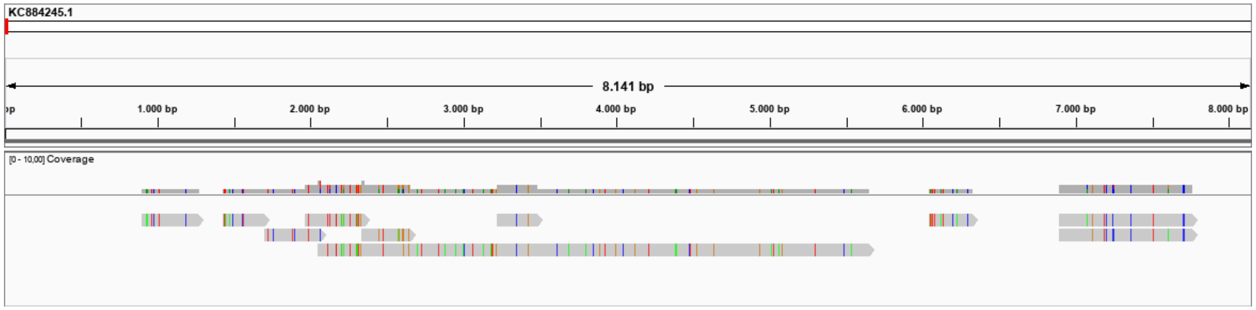


**Figure S1**: Distribution of the assembled transcript on the genome of *Cowpea mild mottle virus* (KC884245.1).Figure as produced using IGV. Colorful lines inside arrows indicate genetic variation in comparison with the reference.

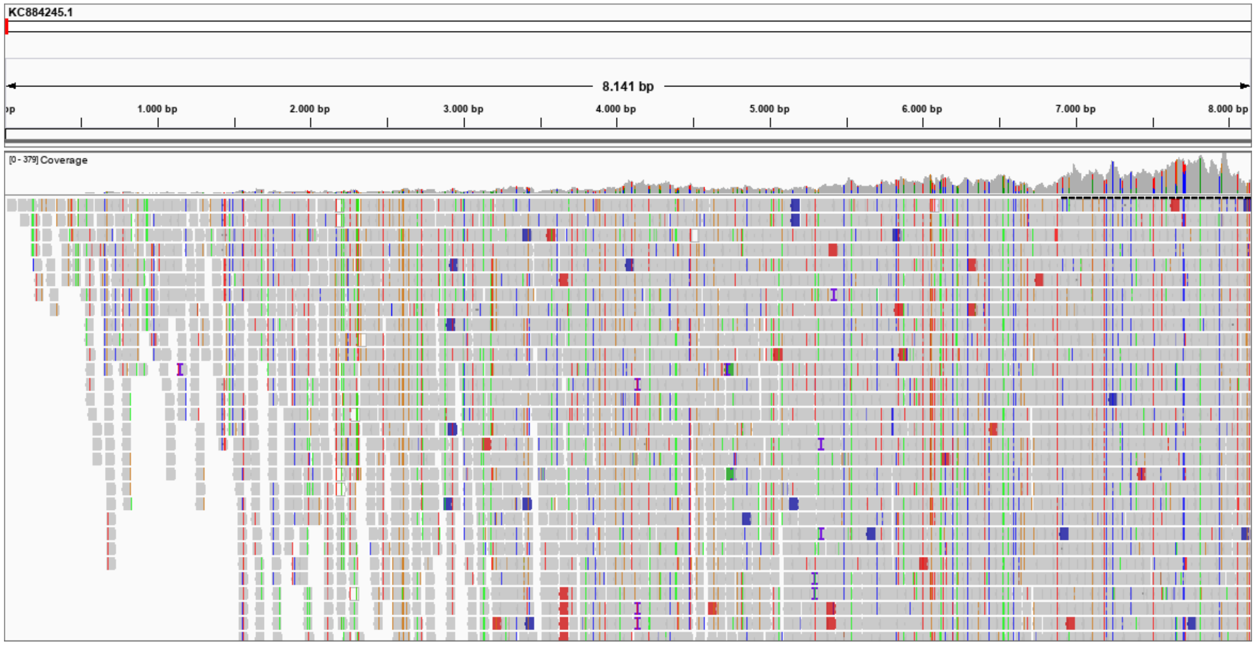


**Figure S2:** Coverage of raw sequencing reads along CPMMV reference genome. Only reads over 50 bp were used in this analysis. The visualization was generated using IGV. Each arrow in the lower part indicates a single sequencing read. In those arrows, colorful regions indicate genetic variation in comparison to the reference. As seen in the middle section, coverage is deeper in the end on the transcript.

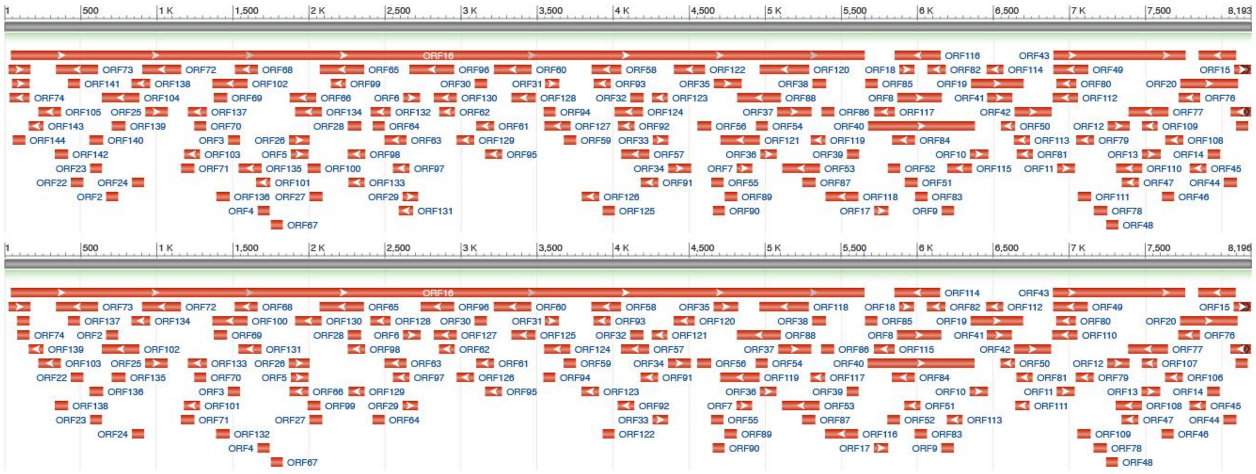


**Figure S3:** Structural annotation of CPPMV strains. ORF profile in CPMMV:BR:MG:09:3 (upper) and CPMMV isolate PB:AF (lower). The visualization was produced using orffinder (https://www.ncbi.nlm.nih.gov/orffinder/) from NCBI portal. The arrow direction indicates the strand which the ORF was identified.

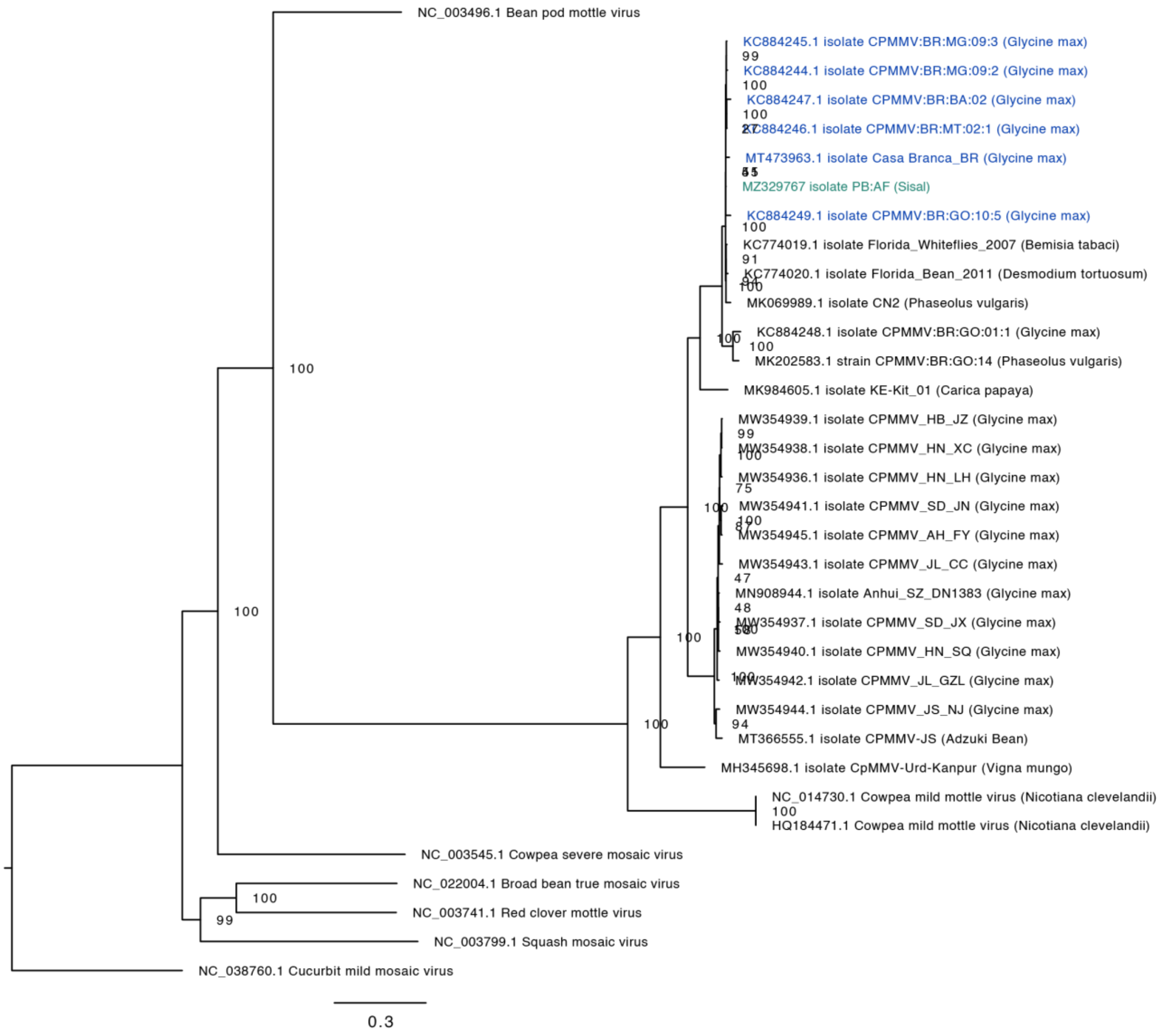


**Figure S4:** Phylogenetic analysis of Cowpea Mild Mottle virus isolate PB:AF. Accession numbers in blue indicate samples from Brazil, while green indicates the isolate assembled in this work.

**Table S8:** The accession codes and descriptions to each viral contig and their respective viral species. “RdRp” estands for RNA-dependent RNA polymerase, and, in this table, is a synonym to “Replicase”.

| Species Name | Contig Name | Acc Code | Protein Type |
|---|---|---|---|
| Cowpea Mild Mottle Virus | Cowpea | MZ329767 | Genome |
| Sisal-associated Alphaflexivirus A | Contig17 | MZ599622 | Putative coat protein |
| Sisal-associated Alphaflexivirus A | TRINITY_DN63052_c0_g1_i1 | MZ599634 | Putative Replicase |
| Sisal-associated Alphaflexivirus B | Contig12 | MZ599625 | Putative Replicase |
| Sisal-associated Alphaflexivirus C | TRINITY_DN38752_c0_g1_i1 | MZ599621 | Putative coat protein |
| Sisal-associated Betaflexivirus A | Contig7 | MZ329756 | RdRp |
| Sisal-associated Betaflexivirus A | TRINITY_DN83885_c0_g1_i1 | MZ599608 | Putative Replicase |
| Sisal-associated Betaflexivirus B | TRINITY_DN88105_c0_g1_i1 | MZ329759 | RdRp |
| Sisal-associated Betaflexivirus B | TRINITY_DN86319_c0_g1_i1 | MZ599609 | Putative Replicase |
| Sisal-associated Betaflexivirus C | TRINITY_DN44239_c0_g1_i1 | MZ329760 | RdRp |
| Sisal-associated Betaflexivirus D | TRINITY_DN52979_c0_g1_i1 | MZ329761 | RdRp |
| Sisal-associated Betaflexivirus D | TRINITY_DN46510_c0_g1_i1 | MZ599640 | Putative Replicase |
| Sisal-associated Betaflexivirus D | TRINITY_DN40814_c0_g1_i1 | MZ599610 | Putative coat protein |
| Sisal-associated Betaflexivirus D | TRINITY_DN65618_c0_g1_i1 | MZ599641 | Putative coat protein |
| Sisal-associated Betaflexivirus E | TRINITY_DN42135_c0_g1_i1 | MZ329762 | RdRp |
| Sisal-associated Botourmiavirus A | TRINITY_DN75741_c0_g1_i1 | MZ599649 | Putative Replicase |
| Sisal-associated Botourmiavirus B | TRINITY_DN110665_c0_g1_i1 | MZ599650 | Putative Replicase |
| Sisal-associated Closterovirus A | TRINITY_DN2548_c0_g1_i1 | MZ329764 | RdRp |
| Sisal-associated Closterovirus A | TRINITY_DN59050_c1_g1_i1 | MZ599642 | hypothetical protein |
| Sisal-associated Closterovirus A | TRINITY_DN49906_c0_g1_i3 | MZ599616 | hypothetical protein |
| Sisal-associated Closterovirus A | Contig16 | MZ599628 | hypothetical protein |
| Sisal-associated Closterovirus A | TRINITY_DN59050_c1_g1_i2 | MZ599643 | hypothetical protein |
| Sisal-associated Closterovirus A | Contig15 | MZ599629 | Putative coat protein |
| Sisal-associated Ribovirus A | TRINITY_DN3617_c0_g1_i1 | MZ599639 | hypothetical protein |
| Sisal-associated Ribovirus A | TRINITY_DN65779_c0_g1_i1 | MZ599646 | Putative Replicase |
| Sisal-associated Unclassified dsRNA virus A | TRINITY_DN81586_c0_g1_i1 | MZ599606 | Putative Replicase |

| | | | |
|---|---|---|---|
| Sisal-associated Unclassified dsRNA virus A | TRINITY_DN61614_c0_g1_i1 | MZ599607 | hypothetical protein |
| Sisal-associated Unclassified dsRNA virus B | Contig14 | MZ599623 | hypothetical protein |
| Sisal-associated Unclassified dsRNA virus B | TRINITY_DN73798_c0_g1_i1 | MZ599644 | Putative Replicase |
| Sisal-associated Unclassified dsRNA virus B | TRINITY_DN116043_c0_g1_i1 | MZ599635 | Putative Replicase |
| Sisal-associated Unclassified dsRNA virus B | TRINITY_DN72309_c0_g1_i1 | MZ599636 | hypothetical protein |
| Sisal-associated Unclassified dsRNA virus C | TRINITY_DN17344_c0_g1_i1 | MZ329765 | RdRp |
| Sisal-associated Unclassified dsRNA virus C | Contig20 | MZ599630 | hypothetical protein |
| Sisal-associated Unclassified dsRNA virus C | Contig1 | MZ599631 | Putative coat protein |
| Sisal-associated Unclassified dsRNA virus C | Contig10 | MZ599632 | Putative coat protein |
| Sisal-associated Unclassified dsRNA virus C | Contig11 | MZ599633 | Putative coat protein |
| Sisal-associated Unclassified dsRNA virus C | TRINITY_DN92324_c0_g1_i1 | MZ599619 | Putative Replicase |
| Sisal-associated Unclassified virus A | TRINITY_DN61147_c0_g1_i1 | MZ599598 | Putative Replicase |
| Sisal-associated Unclassified virus B | TRINITY_DN49676_c0_g1_i2 | MZ329754 | RdRp |
| Sisal-associated Unclassified virus B | TRINITY_DN16142_c0_g1_i1 | MZ599599 | Putative Replicase |
| Sisal-associated Unclassified virus B | TRINITY_DN44352_c0_g1_i1 | MZ599600 | Putative Replicase |
| Sisal-associated Unclassified virus B | TRINITY_DN83710_c0_g1_i1 | MZ599601 | Putative Replicase |
| Sisal-associated Unclassified virus B | TRINITY_DN19804_c0_g1_i1 | MZ599602 | Putative Replicase |
| Sisal-associated Unclassified virus C | TRINITY_DN24880_c0_g1_i1 | MZ329755 | RdRp |
| Sisal-associated Unclassified virus C | TRINITY_DN27401_c0_g1_i1 | MZ599603 | Putative Replicase |
| Sisal-associated Unclassified virus C | TRINITY_DN77293_c0_g1_i1 | MZ599604 | Putative Replicase |
| Sisal-associated Unclassified virus C | TRINITY_DN55189_c0_g1_i1 | MZ599605 | Putative Replicase |
| Sisal-associated Unclassified virus D | Contig19 | MZ599624 | hypothetical protein |
| Sisal-associated Unclassified virus D | TRINITY_DN83752_c0_g1_i1 | MZ599637 | Putative Replicase |
| Sisal-associated Unclassified virus E | DN52810 | MZ329766 | RdRp |
| Sisal-associated Unclassified virus E | TRINITY_DN99951_c0_g1_i1 | MZ599611 | Putative Replicase |
| Sisal-associated Virgavirus A | Contig5 | MZ329757 | RdRp |
| Sisal-associated Virgavirus B | TRINITY_DN100760_c0_g1_i1 | MZ329758 | RdRp |
| Sisal-associated Virgavirus B | TRINITY_DN26278_c0_g1_i1 | MZ599645 | hypothetical protein |
| Sisal-associated Virgavirus B | TRINITY_DN133840_c0_g1_i1 | MZ599638 | hypothetical protein |
| Sisal-associated Virgavirus C | Contig9 | MZ329763 | RdRp |

| | | | |
|---|---|---|---|
| Sisal-associated Virgavirus C | Contig6 | MZ599626 | Putative Replicase |
| Sisal-associated Virgavirus C | TRINITY_DN30831_c0_g1_i1 | MZ599612 | hypothetical protein |
| Sisal-associated Virgavirus C | TRINITY_DN38090_c0_g1_i1 | MZ599620 | Putative coat protein |
| Sisal-associated Virgavirus C | Contig8 | MZ599627 | Putative coat protein |
| Sisal-associated Virgavirus C | TRINITY_DN105616_c0_g1_i1 | MZ599647 | Putative coat protein |
| Sisal-associated Virgavirus C | TRINITY_DN72996_c0_g1_i1 | MZ599613 | Putative Replicase |
| Sisal-associated Virgavirus C | TRINITY_DN26423_c0_g1_i1 | MZ599614 | Putative Replicase |
| Sisal-associated Virgavirus C | TRINITY_DN75281_c0_g1_i1 | MZ599621 | hypothetical protein |
| Sisal-associated Virgavirus C | TRINITY_DN85955_c0_g1_i1 | MZ599615 | Putative Replicase |
| Sisal-associated Virgavirus D | TRINITY_DN83949_c0_g1_i1 | MZ599651 | hypothetical protein |
| Sisal-associated Virgavirus D | TRINITY_DN77371_c0_g1_i1 | MZ599617 | Putative coat protein |
| Sisal-associated Virgavirus D | TRINITY_DN69322_c0_g1_i1 | MZ599618 | hypothetical protein |

**Table S9:** Diversity indices for the organs and species analyzed.

| Index | Leaves AF | Leaves AS | Leaves HI | Stem AF | Stem AS | Stem HI | Roots AF | Roots AS | Roots HI |
|---|---|---|---|---|---|---|---|---|---|
| **Number of Taxa (S)** | 10 | 8 | 4 | 10 | 10 | 11 | 18 | 13 | 12 |
| **Dominance (D)** | 0,3837 | 0,7361 | 0,8649 | 0,3072 | 0,9094 | 0,3317 | 0,401 | 0,2444 | 0,2648 |
| **Simpson Index (1-D)** | 0,6163 | 0,2639 | 0,1351 | 0,6928 | 0,09059 | 0,6683 | 0,599 | 0,7556 | 0,7352 |
| **Shannon Index (H)** | 1,185 | 0,5205 | 0,3017 | 1,414 | 0,2622 | 1,323 | 1,534 | 1,756 | 1,747 |
| **Evenness (e^H/S)** | 0,3271 | 0,2104 | 0,338 | 0,4112 | 0,13 | 0,3413 | 0,2576 | 0,4452 | 0,4782 |
| **Equitability** | 0,5147 | 0,2503 | 0,2176 | 0,6141 | 0,1139 | 0,5516 | 0,5307 | 0,6845 | 0,7031 |

**Table S10:** Viral species and their respective detected protein domains (with Pfam/HMMER) and their expression (in tpm, *transcripts per million*) in each analyzed sample, representing the average expression of the three technical replicates.

| Viral Species | Proteic Domains Detected | Leaves AF | Leaves AS | Leaves HI | Stem AF | Stem AS | Stem HI | Roots AF | Roots AS | Roots HI |
|---|---|---|---|---|---|---|---|---|---|---|
| Sisal-associated Unclassified virus A | none | 1.79 | 0.00 | 0.00 | 0.00 | 0.00 | 0.00 | 0.00 | 0.00 | 0.00 |
| Sisal-associated Unclassified virus B | RdRP, Viral_helicase1 | 0.00 | 0.00 | 0.00 | 0.01 | 0.00 | 0.00 | 87.08 | 0.00 | 0.00 |
| Sisal-associated Unclassified virus C | RdRP, Viral_helicase1, Viral methyltransferase | 0.00 | 0.00 | 0.00 | 0.00 | 0.00 | 0.00 | 30.17 | 0.00 | 0.00 |
| Sisal-associated Alphaflexivirus A | Viral coat protein | 0.00 | 0.00 | 0.32 | 0.00 | 0.56 | 0.09 | 0.00 | 5.65 | 21.86 |
| Sisal-associated Unclassified dsRNA virus A | none | 0.00 | 0.00 | 0.00 | 0.00 | 0.00 | 0.00 | 6.02 | 0.00 | 0.00 |
| Sisal-associated Betaflexivirus A | RdRP | 0.01 | 0.00 | 0.00 | 4.94 | 0.00 | 0.15 | 7.33 | 0.00 | 23.02 |
| Sisal-associated Unclassified dsRNA virus B | none | 0.00 | 0.01 | 0.00 | 0.00 | 0.01 | 1.82 | 7.64 | 15.29 | 4.05 |
| Sisal-associated Unclassified virus D | RdRP | 0.00 | 0.25 | 0.00 | 0.00 | 0.00 | 0.00 | 0.00 | 5.30 | 3.26 |
| Sisal-associated Virgavirus A | none | 0.03 | 0.03 | 0.02 | 0.09 | 0.03 | 0.04 | 23.26 | 30.74 | 34.15 |
| Sisal-associated Alphaflexivirus B | none | 0.00 | 0.00 | 0.00 | 0.00 | 0.00 | 0.00 | 4.09 | 1.62 | 0.00 |
| Sisal-associated Virgavirus B | RdRP, Viral_helicase1 | 0.00 | 0.02 | 0.00 | 0.00 | 0.01 | 0.13 | 0.00 | 13.10 | 4.76 |
| Cowpea Mild Mottle Virus | none | 0.01 | 0.00 | 0.00 | 21.42 | 0.56 | 0.32 | 18.66 | 2.66 | 59.78 |
| Sisal-associated Betaflexivirus B | RdRP | 0.00 | 2.79 | 0.00 | 0.00 | 0.30 | 0.00 | 3.85 | 0.32 | 0.00 |
| Sisal-associated Betaflexivirus C | Viral_helicase1 | 4.35 | 0.00 | 0.00 | 0.55 | 0.00 | 0.00 | 0.55 | 0.00 | 0.00 |
| Sisal-associated Ribovirus A | RdRP, Viral_helicase1 | 0.00 | 0.00 | 0.00 | 0.00 | 0.01 | 0.20 | 0.00 | 13.71 | 3.94 |
| Sisal-associated Betaflexivirus D | Trichovirus coat protein, Viral_helicase1, Viral methyltransferase | 38.15 | 15.98 | 0.00 | 4.97 | 1.30 | 0.00 | 1.14 | 3.11 | 0.00 |

| | | | | | | | | | | |
|---|---|---|---|---|---|---|---|---|---|---|
| Sisal-associated Betaflexivirus E | RdRP | 3.47 | 0.00 | 0.00 | 0.91 | 0.00 | 0.00 | 0.09 | 0.00 | 0.00 |
| Sisal-associated Unclassified virus E | RdRP, RNA helicase | 0.00 | 0.00 | 0.00 | 0.01 | 0.00 | 0.00 | 14.45 | 0.00 | 0.00 |
| Sisal-associated Virgavirus C | DEAD/DEAH box helicase, RdRP, Viral_helicase1, Viral methyltransferase | 1.24 | 0.62 | 1.06 | 3.86 | 0.76 | 1.75 | 407.81 | 106.22 | 151.75 |
| Sisal-associated Alphaflexivirus C | Viral coat protein | 0.00 | 0.00 | 0.00 | 0.00 | 0.00 | 0.00 | 6.59 | 0.00 | 0.00 |
| Sisal-associated Closterovirus A | Closterovirus coat protein, RdRP, Viral methyltransferase | 26.52 | 110.78 | 18.15 | 26.76 | 72.65 | 18.76 | 15.28 | 120.90 | 13.18 |
| Sisal-associated Botourmiavirus A | none | 0.00 | 0.00 | 0.00 | 0.00 | 0.00 | 6.39 | 0.00 | 0.00 | 0.00 |
| Sisal-associated Botourmiavirus B | none | 0.00 | 0.00 | 0.00 | 0.00 | 0.00 | 22.14 | 0.00 | 0.00 | 0.00 |
| Sisal-associated Virgavirus D | none | 0.00 | 0.00 | 0.00 | 0.00 | 0.00 | 0.00 | 14.03 | 0.00 | 4.13 |
| Sisal-associated Unclassified dsRNA virus C | none | 0.07 | 0.00 | 0.00 | 0.00 | 0.00 | 0.00 | 15.95 | 19.12 | 7.53 |
| | **Mean Sequencing Depth** | 117.85 | 131.02 | 107.70 | 152.24 | 131.30 | 107.42 | 141.55 | 124.35 | 118.69 |
